# Ultralow-dose X-ray imaging enabled by vertically homogeneous perovskite films

**DOI:** 10.1093/nsr/nwag391

**Published:** 2026-06-25

**Authors:** Zhiqiang Liu, Jincong Pang, Ziling Zhou, Hao Chen, Qinghao Ling, Chenxing Li, Yuanpeng Shi, Zhuangzhuang Yang, Ling Xu, Zhiping Zheng, Zhen Li, Jiang Tang, Xiaoping Ouyang, Guangda Niu

**Affiliations:** Optical Valley Laboratory, Wuhan 430074, China; Wuhan National Laboratory for Optoelectronics and School of Optical and Electronic Information, Huazhong University of Science and Technology, Wuhan 430074, China; Ezhou Institute of Industrial Technology, Huazhong University of Science and Technology, Ezhou 436044, China; Optical Valley Laboratory, Wuhan 430074, China; Wuhan National Laboratory for Optoelectronics and School of Optical and Electronic Information, Huazhong University of Science and Technology, Wuhan 430074, China; Ezhou Institute of Industrial Technology, Huazhong University of Science and Technology, Ezhou 436044, China; Department of Radiology, Tongji Hospital, Tongji Medical College, Huazhong University of Science and Technology, Wuhan 430030, China; Department of Radiology, Tongji Hospital, Tongji Medical College, Huazhong University of Science and Technology, Wuhan 430030, China; Wuhan National Laboratory for Optoelectronics and School of Optical and Electronic Information, Huazhong University of Science and Technology, Wuhan 430074, China; Wuhan National Laboratory for Optoelectronics and School of Optical and Electronic Information, Huazhong University of Science and Technology, Wuhan 430074, China; Wuhan National Laboratory for Optoelectronics and School of Optical and Electronic Information, Huazhong University of Science and Technology, Wuhan 430074, China; School of Integrated Circuits, Huazhong University of Science and Technology, Wuhan 430074, China; Wuhan National Laboratory for Optoelectronics and School of Optical and Electronic Information, Huazhong University of Science and Technology, Wuhan 430074, China; School of Integrated Circuits, Huazhong University of Science and Technology, Wuhan 430074, China; Wuhan National Laboratory for Optoelectronics and School of Optical and Electronic Information, Huazhong University of Science and Technology, Wuhan 430074, China; School of Integrated Circuits, Huazhong University of Science and Technology, Wuhan 430074, China; Department of Radiology, Tongji Hospital, Tongji Medical College, Huazhong University of Science and Technology, Wuhan 430030, China; Optical Valley Laboratory, Wuhan 430074, China; Wuhan National Laboratory for Optoelectronics and School of Optical and Electronic Information, Huazhong University of Science and Technology, Wuhan 430074, China; Northwest Institute of Nuclear Technology, Xi’an 710024, China; Optical Valley Laboratory, Wuhan 430074, China; Wuhan National Laboratory for Optoelectronics and School of Optical and Electronic Information, Huazhong University of Science and Technology, Wuhan 430074, China; Ezhou Institute of Industrial Technology, Huazhong University of Science and Technology, Ezhou 436044, China; Department of Radiology, Tongji Hospital, Tongji Medical College, Huazhong University of Science and Technology, Wuhan 430030, China; Research Institute of Huazhong University of Science and Technology in Shenzhen, Shenzhen 518057, China

**Keywords:** ultralow photon fluxes, low-dose X-ray imaging, liquid-phase growth and annealing, vertical uniformity

## Abstract

Artificial intelligence is rapidly pushing imaging and sensing toward automated, quantitative decision-making, heightening the demand for reliable low-photon-flux detection and imaging across infrared through X-ray regimes, yet developing detectors that truly operate effectively at ultralow photon fluxes has been a challenge. As a canonical instance, low-dose X-ray imaging operates under intrinsically sparse photon statistics, where Poisson fluctuations along the absorption depth couple to vertical transport non-uniformity and are statistically amplified at ultralow dose. Here we elucidate this materials-to-electronics bottleneck and develop a new liquid-phase growth and annealing strategy that eliminates thermal and mass-transport instabilities in conventional methods during crystallization, yielding perovskite films with exceptional vertical uniformity. This enables depth-independent charge collection, significantly reducing stochastic fluctuations in the readout and achieving a 10-fold reduction in image noise. In imaging applications, our detectors deliver high-quality X-ray imaging at an ultralow effective per-pixel integration dose of 40.6 nGy_air_, setting a new benchmark for safe, high-quality clinical imaging.

## Teaser

This work shows that using more evenly structured perovskite films in X-ray detectors greatly reduces image noise, enabling clear medical images at much lower radiation doses and supporting safer AI-assisted screening.

## INTRODUCTION

Artificial intelligence (AI) is rapidly reshaping X-ray imaging from manual visual inspection to automated, quantitative decision-making [1]. By reducing interpretation burden and enabling standardized readouts, AI-assisted workflows are accelerating large-scale screening, long-term monitoring, and follow-up examinations, with clear benefits for early diagnosis and treatment planning [2,3]. At the same time, the rising volume of clinical imaging increases cumulative radiation exposure, raising concerns about radiation-induced genetic damage and cancer risk, and therefore intensifies the demand for reliable imaging at substantially reduced dose [4,5]. Most commercial systems use scintillator–silicon detectors, which suffer substantial signal loss due to indirect conversion, resulting in total sample entrance dose as high as ∼1–4 μGy_air_ per frame [6,7]. As a result, a single modern digital radiography (DR) or computed tomography (CT) scan can deliver an effective dose up to 8.0 mSv—equivalent to 1–3 years of natural background radiation (2.4 mSv per year) [8,9].

Direct-conversion semiconductors such as amorphous selenium (a-Se) and emerging halide perovskites offer higher X-ray responsivity and, in principle, should enable lower-dose operation [10,11]. However, in practice, these materials underperform even commercial FPDs. For instance, a-Se detectors require high doses of 9–15 μGy_air_ [6,12], and halide perovskites—despite responsivities orders of magnitude higher than scintillators [13]—demand total sample entrance dose as high as ∼25 μGy_air_ per frame due to severe electronic noise [14–16]. Recent efforts to suppress noise *via* compositional engineering of perovskite crystals have shown promise, but remain restricted to millimeter-scale devices, far from the large-area formats essential for clinical or industrial imaging [17–19]. As such, realizing large-area, high-responsivity and low-noise detectors for true low-dose imaging remains an unresolved challenge.

**Figure 1.**
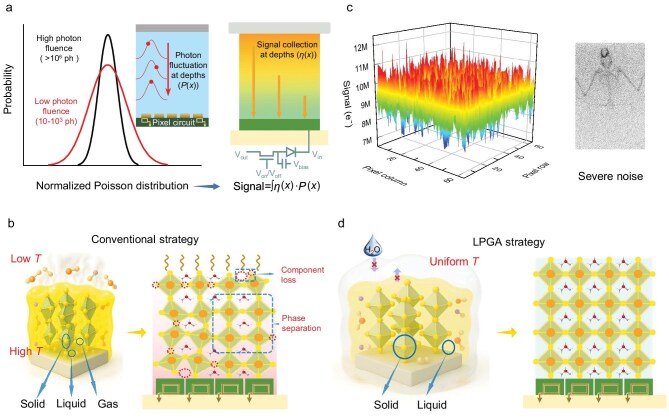
Impact of Poisson fluctuations in photon deposition and vertical uniformity on low-dose imaging. (a) Schematic illustration of photon fluence-dependent Poisson fluctuations and the impact of vertical non-uniformity in perovskite films on signal collection. (b) Schematic illustration of perovskite thick-film fabrication in a solid-liquid-gas three-phase system. (c) Influence of vertical crystallinity uniformity on image signal-to-noise ratio and noise level. (d) Schematic illustration of perovskite thick-film fabrication in a solid-liquid two-phase system.

In this work, we identify detector vertical uniformity—a long-overlooked factor in X-ray detectors—as a critical factor for suppressing readout noise in large-area perovskite detectors (Fig. 1a). Unlike visible or infrared detectors, X-ray detectors operate with 4–6 orders of magnitude fewer incident photons and necessitate much thicker active layers (hundreds of micrometers), making the signal highly susceptible to vertical inhomogeneity and noise accumulation (see main text for derivations).

Prior fabrication techniques—such as blade-coating, spray deposition and thermal annealing—typically involve complex transitions among precursor solution (liquid), volatile solvents or annealing atmospheres (gas), and crystallized perovskites (solid) [14,20]. These uncontrolled liquid-gas-solid interactions, along with chaotic local temperature and ambient variations, often result in highly non-uniform crystal growth (Fig. 1b). Even within a single vertically continuous grain, such unstable processing can introduce intra-grain inhomogeneities [21]. While these vertical non-uniformities have already been observed in sub-micron photovoltaic films [22,23], they become far more pronounced and detrimental in the hundred-micrometer-thick layers required for X-ray detection, severely degrading carrier transport and amplifying electronic noise.

To address this problem, we propose and demonstrate a fully liquid-phase growth and annealing (LPGA) strategy for perovskite imager fabrication. By eliminating gas-phase involvement and leveraging a controlled liquid-solid phase transition, this approach ensures uniform local temperature and atmosphere throughout the film, thereby avoiding the vertical non-uniformities inherent to conventional fabrication methods. The resulting perovskite detectors exhibit a 10-fold reduction in noise (image noise down to 62090 e^−^). In imaging applications, our devices achieve a high detective quantum efficiency of 71.6% under an ultralow effective per-pixel integration dose of 40.6 nGy_air_, enabling high-contrast imaging of a molar tooth, bat skeleton, and walnut, and setting a new benchmark for safe, high-quality clinical imaging. More broadly, such ultralow-dose imaging provides a sensor-level foundation for scalable, AI-enabled diagnosis, early screening, and population-level examinations.

## RESULTS

We first conducted physical modeling and numerical simulations to evaluate how vertical non-uniformity contributes to image noise in flat-panel X-ray detectors. In X-ray detection, incident photons arrive at the detector as discrete particles governed by Poisson statistics, where the standard deviation in photon count equals the square root of the mean [24]. At high photon flux—on the order of 10^6^–10^8^ photons per pixel, as in visible-light imaging—the relative fluctuation (<0.1%) is negligible. However, under typical clinical X-ray dose (1.25 μGy_air_), only ∼377 photons are detected per 100 μm pixel, resulting in >5% statistical fluctuation (Fig. 1a and Note S1) [25]. Under the ultralow-dose conditions targeted in this study, photon counts drop by another 1–2 orders of magnitude, further exacerbating stochastic variation (>17%) in the depth distribution of absorption events.

This increased fluctuation breaks the smooth exponential absorption profile predicted by the Beer–Lambert law, leading to pronounced intrinsic pixel-to-pixel variability. For example, in a 500 μm-thick perovskite film under the low-dose conditions discussed in this work (∼10 photons per pixel), the law predicts ∼8 photons absorbed in the top 250 μm and ∼2 in the bottom 250 μm for every pixel. However, Poisson-governed statistics can cause large deviations for individual pixels (e.g. 6 and 4 photons in the respective regions, Note S2). This fluctuation is an intrinsic physical process and cannot be eliminated. In an ideal film with perfectly uniform vertical transport, all absorbed photons—regardless of depth—contribute equally to charge collection. In this case, stochastic fluctuations (including both Poisson and binomial statistics) cancel out statistically, exerting negligible influence on signal uniformity (Fig. S1). However, achieving such an ideal detector is extremely challenging, particularly for thick X-ray absorber layers. Any vertical non-uniformity—such as variations in trap density, crystal quality, or carrier mobility–lifetime product—leads to depth-dependent charge collection efficiency, causing photons absorbed at different depths to contribute unequally to the output signal. Consequently, stochastic fluctuations in photon deposition depth become amplified rather than averaged out, manifesting as pixel-to-pixel readout noise and degrading image quality (Fig. 1c).

To quantify this effect, we performed simulations in which the film was segmented vertically, with each layer assigned a depth-dependent charge collection efficiency (Note S2). As expected, the resulting flat-panel output exhibited substantial image noise under low-photon-flux conditions, with intensity variation reaching ∼700000 e^−^ (Fig. S2). This matched experimental results for conventional polycrystalline perovskite detectors (Fig. S3). This also explains why previous efforts focusing solely on lateral film uniformity and integration interface have failed to effectively eliminate image noise in perovskite X-ray detectors [26,27]. The combination of intrinsic Poisson-limited photon statistics and vertically non-uniform charge transport forms a fundamental barrier to low-dose, high-fidelity imaging.

**Figure 2.**
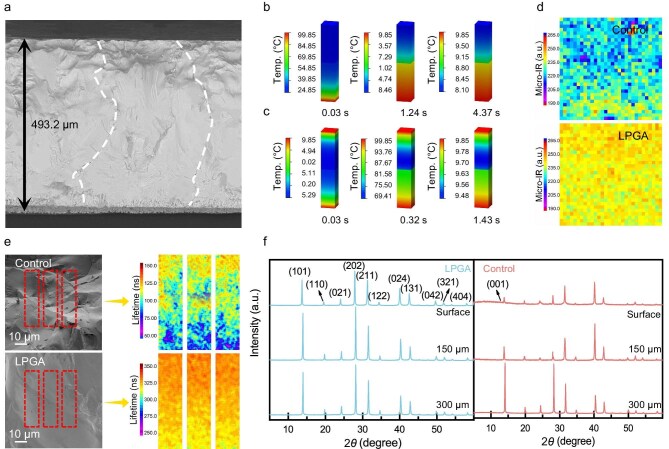
Characterization of the vertical uniformity in thick perovskite films. (a) Cross-sectional SEM image of the perovskite thick film prepared by the LPGA method. (b, c) Finite element analysis of the temporal evolution of the thermal field distribution for (b) the control sample and (c) the LPGA-treated sample. (d) Micro-infrared (IR) mapping of the perovskite thick film at 1480 cm^−1^, corresponding to the C–N stretching vibration of MA^+^ ions. (e) Time-resolved photoluminescence (TRPL) mapping of the cross-sections of the control and LPGA-treated samples. (f) X-ray diffraction (XRD) patterns of the control and LPGA samples at different depths.

To overcome the vertical inhomogeneity described above, we developed a fully LPGA strategy that completely avoids the unstable liquid-gas-solid transitions intrinsic to conventional fabrication methods (Fig. 1d). By eliminating gas-phase involvement, LPGA ensures a uniform thermal and chemical environment throughout both crystallization and annealing, effectively suppressing depth-dependent variations in crystallinity. Beyond the present X-ray detectors, this concept is broadly relevant to perovskite optoelectronics, where vertical uniformity is known to be pivotal for photovoltaic efficiency and LED stability [22,28,29]. However, prior approaches typically rely on complex intermediate-phase control or anion–π interactions and are restricted to tens-to-hundreds-nanometer films [21,23,30]. In contrast, LPGA can produce perovskite films with millimeter-thickness and excellent depth uniformity, improving thickness scalability by three orders of magnitude. Given that the difficulty of vertical control escalates sharply with thickness, achieving bulk-scale homogeneity, to our knowledge, represents a significant materials advance. Specifically, the process begins with chemical bath deposition (CBD), wherein the perovskite precursor solution gradually crystallizes within a temperature-controlled solvent medium. This promotes steady nucleation and vertical grain growth, free from solvent evaporation or air interference (Fig. S4). Using this approach, we successfully fabricated dense polycrystalline thick films (∼500 μm) composed of vertically aligned grains that span the full film thickness (Fig. 2a). In contrast, all previously reported perovskite detectors, as well as commercial detectors based on cadmium zinc telluride, typically exhibit vertically segmented grain structures, which can hinder charge transport across grains and introduce noise [14,15,31].

Subsequently, instead of annealing the film on a hotplate—where vertical thermal gradients and gas-phase perturbations are inevitable—the as-deposited films are transferred into a nonpolar oil medium and annealed at elevated temperatures (typically 80–120°C). The oil medium simultaneously extracts residual solvents, remains chemically inert to perovskite, and facilitates rapid and uniform heat transfer. In this work, we selected anisole as the oil medium, owing to its high thermal conductivity (0.137  W m^−1^ K^−1^), low viscosity (0.99  mPa·s at 20°C), and strong compatibility with common perovskite solvents (e.g. GBL, DMF, DMSO) (Fig. S5). XRD confirmed that PbI_2_·DMSO adducts were fully removed after 30 s anisole annealing at 100°C, whereas they persisted in hotplate-treated films (Fig. S6a). To more directly evaluate the capability of the LPGA process to extract residual solvent from the perovskite thick film, we performed thermogravimetric analysis on thick films prepared by conventional hotplate annealing and by LPGA annealing (Fig. S6b, c). The hotplate annealing thick film exhibited an additional weak and broad low-temperature weight loss peak at ∼180–220°C, with a mass loss of only about 1–2 wt%, while this peak was not observed in the LPGA-treated film. Considering that GBL has a boiling point of ∼202–204°C, this low-temperature signal is more reasonably explained by the volatilization/release of a small amount of residual GBL, rather than the decomposition of the perovskite framework itself. In contrast, the main thermal decomposition of the perovskite framework occurs at higher temperatures (∼320–370°C). These results further support that LPGA is more effective in removing residual GBL from the thick perovskite film.

To compare the thermal environments, we performed finite element simulations of annealing in the LPGA versus traditional fabrication process (denoted as the ‘control’) (Note S3). Hotplate annealing induces a 1.7°C vertical temperature gradient across the 500 μm-thick film, requiring 4.4 s to reach equilibrium (Fig. 2b, c). In contrast, the LPGA process achieves thermal equilibrium within 1.4 s with only 0.4°C vertical difference, enabled by isotropic heat transfer in the oil. The oil medium also suppresses convective heat loss to air, further stabilizing the annealing environment (Fig. S7a). An added advantage of the LPGA strategy is that all operations can be performed in ambient air. The liquid medium shields the film from air-borne degradation during annealing, minimizing undesirable reactions between volatile species (e.g. MA^+^ and I^−^) and ambient air (e.g. H_2_O, O_2_) (Fig. S7b). Micro-IR analysis of the C–N stretching band at 1480 cm^−1^ revealed stronger, more localized MA^+^ signals in LPGA films (Fig. 2d), suggesting reduced volatilization. XPS analysis further confirmed improved stoichiometry, with the I/Pb ratio in LPGA films approaching the ideal value of 3 (Fig. S8), indicating suppression of surface degradation and defect formation.

To more directly probe electrically active traps, we further performed thermally stimulated current (TSC) measurements (Fig. S9). Compared with the control film, the LPGA film exhibited a substantially reduced integrated TSC response. The broad TSC feature observed in the thick films is likely associated with the superposition of multiple trap contributions originating from grain boundaries, surfaces, interfaces, and bulk defects, rather than from only a few discrete trap families. In particular, the corresponding feature in the control film is broader and more intense, whereas that in the LPGA film is evidently weakened. These results demonstrate that LPGA suppresses the formation of electrically active traps in thick perovskite absorber layers, consistent with the reduction in trap-assisted recombination, lower dark current, and improved operational stability observed in device measurements.

Cross-sectional TRPL mapping and depth-resolved XRD showed that LPGA films exhibit superior vertical uniformity in carrier lifetime and crystallinity compared to control samples. Notably, the LPGA film displayed significantly longer and more consistent carrier lifetimes (300–350 ns) compared to control samples (50–150 ns) (Fig. 2e), as well as vertically consistent crystallinity (Fig. 2f), with the main diffraction peaks indexed to the cubic α-FAPbI_3_ phase. Control films showed surface-segregated PbI_2_ at 12.3°, while LPGA samples maintained phase purity throughout the film. Enhanced PL intensity (Fig. S10a) and reduced trap-assisted recombination (Fig. S10b) further underscore the superior vertical and lateral uniformity enabled by the LPGA approach. Additional wide-field cross-sectional TRPL mapping further distinguishes lateral and vertical variations within the same cross-sectional window, showing that the control film is relatively uniform laterally but strongly non-uniform through the thickness, whereas the LPGA film is uniform in both directions (Note S4 and Fig. S10c, d).

To evaluate the practical impact of vertical film uniformity, we fabricated thick-film X-ray detectors using both LPGA-grown films and control films, and systematically compared their electronic and X-ray response characteristics. Both devices adopted a vertical photoconductor configuration with Au top electrodes and ITO bottom contacts. The film thicknesses were kept consistent (∼500 μm) to eliminate thickness-related variability. At this thickness, ∼96% of incident photons are absorbed under RQA3 X-ray spectrum (mean energy = 42.6 keV; Fig. S11). We first analyzed the detection efficiency (DE), which reflects the combined photon absorption and charge collection efficiency. The DE was calculated using the *μτE* model (Fig. S12). Below 500 μm, DE is mainly limited by insufficient photon absorption, but beyond this point, charge collection efficiency begins to decline due to increased charge recombination. Hence, the 500 μm thickness represents an optimal trade-off, yielding a high DE of 89.7% (Note S5).

**Figure 3.**
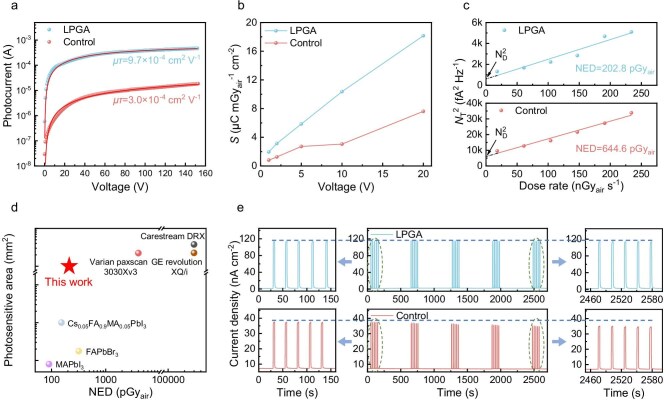
Electrical properties of the control and LPGA perovskite thick-film devices. (a) Bias-dependent photocurrent. The data were fitted using the Hecht equation. (b) Sensitivity at different bias voltages. (c) Dependence of squared noise current spectral density on the dose rate. (d) Comparison of photosensitive areas and NED values for representative X-ray detectors. (e) X-ray irradiation stability of unencapsulated devices.

Steady-state photocurrents under varying bias voltages were fitted using a modified Hecht equation to extract the carrier mobility-lifetime product *μτ* [32]. The LPGA device exhibited a markedly higher *μτ* value of 9.7 × 10^−4^ cm^2^ V^−1^, compared to 3.0 × 10^−4^ cm^2^ V^−1^ for the control (Fig. 3a). Time-of-flight measurements further confirmed excellent charge transport in LPGA devices, with hole and electron mobilities of 63.3 and 105.6 cm^2^ V^−1^ s^−1^, respectively (Fig. S13), far exceeding the values typically observed in polycrystalline perovskite thick films and approaching those of high-quality single crystals [33,34]. This remarkable performance is attributed to the vertically aligned grains formed *via* LPGA, which minimize grain boundaries along the drift path and mimic single-crystal behavior.

We next evaluated X-ray response under dose rates of 28–113 μGy_air_ s^−1^. The LPGA device exhibited highly linear photocurrent behavior (Fig. S14) and significantly higher response than the control. At applied biases of 1, 2, 5, 10, and 20 V, the LPGA detector achieved sensitivities of 1.95 × 10^3^, 3.13 × 10^3^, 5.86 × 10^3^, 1.03 × 10^4^, and 1.81 × 10^4^ μC Gy_air_^−1^ cm^−2^, respectively (Fig. 3b). Additionally, it exhibited lower dark current, which helps suppress thermal and shot noise [35]. We further extracted the bulk resistivity from the dark current-voltage characteristic curves (Fig. S15). The resistivity of the LPGA film (∼1.23 × 10^9^ Ω⋅cm) was higher than that of the control film (∼7.01 × 10^8^ Ω⋅cm). This result is consistent with its lower residual solvent content and fewer electroactive leakage paths. To further evaluate ionic transport behavior, we performed temperature-dependent conductivity measurements and analyzed the Arrhenius relation of ln(*σT*) versus 1000/*T* (Fig. S16). Compared with the control, the LPGA device exhibited a larger apparent activation energy of ionic migration (∼0.42 eV versus ∼0.26 eV) and a higher onset temperature for ionically dominated conduction (∼290 K versus ∼255 K). These results indicate that ionic motion is more strongly suppressed after LPGA treatment, consistent with the reduced dark-current drift and improved long-term operational stability of the LPGA device.

Transient photocurrent measurements revealed a fall time of 1.8 ms for the LPGA device versus 4.1 ms for the control (Fig. S17), indicating reduced carrier trapping and enabling shorter exposure durations for safer imaging. We further mapped the spatial response across eight device regions (Fig. S18a, b), with the LPGA sample showing minimal variation in both dark current and photocurrent (Fig. S18c). Reduced and stable dark current minimizes dark noise, while consistent and strong photocurrent improves signal strength and mitigates readout and 1/*f* noise, resulting in a superior signal-to-noise ratio under low-dose conditions.

The noise equivalent dose (NED) quantifies the minimum detectable dose limited by the detector’s intrinsic noise. We measured the X-ray dose-rate-dependent noise spectral density under 5 V bias (Note S6), and the noise current spectral density ranged from 10^−13^ to 10^−14^ A Hz^−1/2^. From these measurements, the NED—expressed as the equivalent photon quantity—was extracted by fitting the dose-dependent noise current. The LPGA device exhibited significantly lower noise than the control (Fig. S19), yielding an NED of 202.8 pGy_air_, corresponding to ∼30 photons at 42.6 keV (Fig. 3c). To contextualize this performance, we constructed a detector performance map comparing NED versus effective detection area across various technologies (Fig. 3d). While several perovskite single crystal detectors (e.g. Cs_0.05_FA_0.9_MA_0.05_PbI_3_, MAPbI_3_, FAPbBr_3_) have reported similarly excellent NEDs (∼100–300 pGy_air_), their active areas remain limited to the mm^2^ scale. In contrast, our LPGA device sustains ultralow NED at the dm^2^ scale (Fig. S20) [17,36,37]. By comparison, commercial direct and indirect flat-panel detectors (e.g. Carestream DRX, Varian PaxScan 3030Xv3, GE Revolution XQ/i) operate over dm^2^ areas but exhibit NEDs in the nGy_air_ range or higher—2–4 orders of magnitude above our device. Long-term operational stability was assessed by monitoring the dark current drift over 1 h. The LPGA device demonstrated a drift rate of only 9.7 × 10^−7^ nA cm^−1^ s^−1^ V^−1^—on par with or better than state-of-the-art single-crystal detectors (Fig. S21) [38]. Additionally, the LPGA device maintained a stable photocurrent across 2750 s of repeated X-ray switching, while the control device exhibited significant performance degradation (Fig. 3e). This enhanced stability is attributed to the removal of residual solvents and suppression of volatile loss during annealing. Collectively, these results demonstrate that LPGA-fabricated perovskite detectors combine improved trap suppression, higher resistivity, reduced ionic instability, excellent charge transport, low noise, and enhanced operational stability, while maintaining area scalability.

We next assessed the low-dose imaging performance of the LPGA perovskite detectors using a monolithic 64 × 64 thin-film transistor (TFT) array (200 μm pixel pitch, 12.8 mm × 12.8 mm effective area; Fig. S22). Unlike previously reported single-pixel scanning schemes, our full-array architecture enables direct integration with automatic signal readout—crucial for real-time clinical DR.

**Figure 4.**
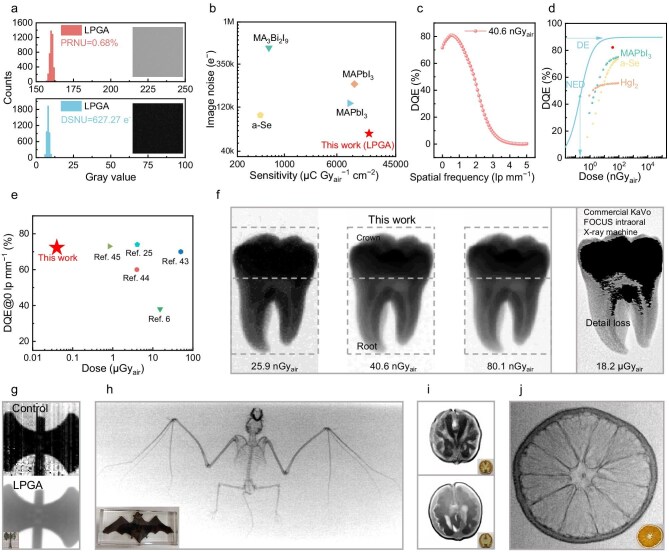
X-ray imaging performance. (a) Pixel grayscale value distribution of all pixels of the LPGA device under X-ray irradiation and dark conditions, and the inset is the corresponding grayscale value image. (b) Comparison of sensitivity and image noise among various detectors. (c) The relationship between the DQE curve of X-ray FPDs measured at an effective per-pixel integration dose of 40.6 nGy_air_ and the spatial frequency. (d) DQE curve dependence on the X-ray dose. (e) Comparison of imaging dose and DQE of representative X-ray flat-panel detectors. (f) X-ray images of a single mandibular molar. The first three columns correspond to images acquired with our LPGA device, while the fourth column shows the image obtained using a commercial KaVo FOCUS intraoral X-ray system. (g) X-ray images of metal axes using LPGA and control FPDs at an effective per-pixel integration dose of 40.6 nGy_air_. X-ray images of (h) a bat, (i) walnut slices and (j) an orange slice captured by LPGA FPDs under an effective per-pixel integration dose of 40.6 nGy_air_.

It is important to note that the noise behavior of such integrated arrays differs fundamentally from that of single-pixel detectors. In addition to spectral noise originating from charge transport and readout, array-level noise also includes fixed-pattern noise stemming from pixel-to-pixel non-uniformity. To assess these contributions, we evaluated pixel-wise photo-response non-uniformity (PRNU) and dark signal non-uniformity (DSNU) under X-ray and dark conditions, respectively (Note S7). The LPGA device exhibited a PRNU of 0.68% (Fig. 4a), far lower than the control device (8.05%) and superior to existing a-Se (<5%), HgI_2_ (10%), and CdZnTe (20%) detectors (Fig. S23a, b) [39,40]. Similarly, DSNU was reduced from 5401 e^−^ (Fig. 4a) to 627 e^−^, placing our device among the lowest reported noise levels for large-area imagers (Fig. S23c, d). Together, these metrics demonstrate outstanding pixel uniformity and reduced fixed-pattern noise in the integrated array. Stacked-frame analysis further allowed us to separate temporal random noise from spatial fixed-pattern noise in both dark and illuminated images (Fig. S23e, f). In both cases, the LPGA device shows lower temporal and spatial noise than the control, but the improvement in the fixed-pattern component is much more pronounced.

Furthermore, we systematically evaluated the total noise of the detector. It is worth noting that, although several studies in the field of perovskite X-ray imaging have reported low noise and low-dose imaging using either single-pixel scanning detectors or devices without fully pixelated readout, such measurements typically rely on high-end instrumentation equipped with built-in noise suppression functions (e.g. high-sensitivity electrometers) [17,36]. As a result, their reported performance does not faithfully reflect the intrinsic noise level and practical capability of a complete imaging system. In real-world applications, detector noise arises from the superposition of multiple contributions—including the intrinsic device noise, pixel-to-pixel variations, and the integrated readout circuitry. Consequently, measurements based on non-array devices may significantly underestimate the inherent noise of the detector. To ensure a fair comparison, we restricted our benchmark to detectors featuring array-level integration and automatic signal readout, thereby capturing the true noise characteristics and imaging performance of the entire system. Compared with the control devices, the LPGA detectors exhibited more than an order-of-magnitude reduction in image noise (62090 e^−^ versus 751041 e^−^), outperforming not only existing perovskite-based detectors (e.g. MAPbI_3_: 131682 e^−^, MAPbI_3_: 213004 e^−^, MA_3_Bi_2_I_9_: 524595 e^−^) but also state-of-the-art commercial DR systems (e.g. a-Se: 98000 e^−^) (Fig. 4b and Note S7) [14,15,41,42].

To quantify overall imaging fidelity under low-dose conditions, we calculated the detective quantum efficiency (DQE), defined as:


\begin{eqnarray*}
{\mathrm{DQE}}\left( f \right) = \frac{{q \times {{G}^2} \times {\mathrm{MT}}{{{\mathrm{F}}}^2}\left( f \right)}}{{{\mathrm{NPS}}\left( f \right)}},
\end{eqnarray*}

where MTF(*f*) is the modulation transfer function, NPS(*f*) is the noise power spectrum, and *q* is the incident photon fluence per unit dose. A detailed explanation of the dose estimation and DQE calculation procedure is provided in Note S8. To ensure clinical relevance, the X-ray beam was calibrated according to the IEC 61267:1994 RQA3 standard using aluminum filtration and an ionization chamber dosimeter. For the TFT-based pixel array, which operates in a row-by-row rolling-readout mode, the effective per-pixel integration dose was determined by dividing the total dose rate by the frame rate and the number of pixel rows. Under a dose rate of 28.32 μGy_air_ s^−1^, a frame rate of 10.88 fps, and 64-row sequential readout, the row activation time was 1/(10.88 × 64) s, corresponding to an effective per-pixel integration dose of 40.6 nGy_air_. In the present continuous-illumination prototype, the total sample entrance dose per full-frame acquisition was 28.32/10.88 = 2.60 μGy_air_. The spatial resolution was evaluated using the slanted-edge method (Note S9). The resulting modulation transfer function (MTF) reached 2.75 lp mm^−1^ at 20% contrast (Fig. S24a), among the best values reported for clinical DR systems (e.g. GE Revolution XQ/i: 2.6 lp mm^−1^; Varian PaxScan 4030: 2.5 lp mm^−1^). Given the 200 μm pixel pitch of our TFT array, this spatial resolution approaches the theoretical limit and is sufficient for clinical diagnostics, which prioritize lesion contrast over ultra-fine spatial details. Meanwhile, the NPS exhibited a steep decay with increasing spatial frequency, indicating effective suppression of noise contributions across the imaging bandwidth (Fig. S24b).

Under an effective per-pixel integration dose of 40.6 nGy_air_, the LPGA detector achieved an unprecedented high DQE(0) of 71.6%, with the DQE(*f*) curve maintaining high values across spatial frequencies (Fig. 4c). Theoretical DQE, which was derived from the independently measured NED and DE, closely matched the experimental DQE (red dot in Fig. 4d), confirming that minimal additional fixed-pattern noise was introduced by the integration. We benchmarked our detector against commercial DR systems and state-of-the-art detectors. As shown in Fig. 4e, the LPGA detector significantly outperforms commercial platforms. Specifically, the LPGA detector achieves 71.6% DQE at an effective per-pixel integration dose of 40.6 nGy_air_, compared to 64% at 2.35 μGy_air_ (GE Revolution XQ/i), 38% at 15.13 μGy_air_ (Hologic DR-1000) and 75% at 103 μGy_air_ (Fujifilm Amulet) [6,25,43–45]. Notably, compared to reported perovskite and inorganic detectors—such as MAPbI_3_ (75.3% at 525.9 μGy_air_) and CdZnTe (70% at 278 μGy_air_)—our detector uniquely combines ultralow-dose operation with high DQE over a clinically relevant area [20,46]. A more detailed comparison is shown in Table S3. For clarity, the value reported for this work refers to the pixel-level effective integration dose rather than the total sample entrance dose per full-frame acquisition.

To validate the practical imaging capability, we acquired dental X-ray images of a molar at multiple effective per-pixel integration doses of 25.9, 40.6, and 80.1 nGy_air_ (Fig. 4f). Remarkably, even at the lowest effective per-pixel integration dose of 25.9 nGy_air_, the LPGA detector clearly resolved the details of the pulp cavity in the crown region and the dentin in the root region. In contrast, imaging the same tooth with a commercial KaVo FOCUS intraoral X-ray system, even at a much higher dose of 18.2 μGy_air_, resulted in poor dentin contrast and blurred structural features. This demonstrates that our detector achieves superior image quality relative to commercial intraoral systems, even with a significant reduction in radiation dose. This level of dose reduction is particularly attractive for dose-sensitive applications such as digital subtraction angiography and pediatric or prenatal CT, where cumulative radiation exposure is a significant concern. Further imaging of a metal axe verified superior edge sharpness and uniformity of the LPGA device (Fig. 4g). Finally, stitched imaging of a bat skeleton (Fig. 4h), walnut slices (Fig. 4i), and an orange slice (Fig. 4j) revealed delicate structural details, confirming suitability for biological and industrial nondestructive imaging at ultralow dose.

We note that the present prototype was developed to evaluate detector-level low-dose imaging capability under row-selective current-mode readout, rather than to minimize the cumulative sample/patient entrance dose in a clinically synchronized imaging configuration. In the current architecture, the X-ray source remains on during full-frame acquisition, while photogenerated charge is stored in external capacitors integrated in the analog-to-digital converter (ADC) and read out sequentially row by row. Consequently, this design is suitable for assessing detector performance, but does not yet represent the optimal system-level solution for reducing patient exposure in practical clinical imaging. The central result of this work is that the LPGA-fabricated detector can sustain high DQE at an effective per-pixel integration dose of 40.6 nGy_air_, highlighting its strong potential for dose reduction. Future translation to lower patient exposure will require a globally integrating TFT backplane with in-pixel storage capacitance synchronized to a short pulsed X-ray source, so that exposure occurs only during a brief integration window and the stored charge is read out afterward with the X-ray source turned off.

## CONCLUSION AND OUTLOOK

In this work, we identify and validate a previously overlooked intrinsic constraint in X-ray detection: the inherently sparse photon flux imposes strong Poisson statistics that propagate along the vertical absorption pathway of thick active layers, amplifying noise under ultralow-dose conditions. This principle, while revealed here in the context of X-ray imaging, has broad implications for low-photon flux detection platforms including photon-counting detectors, particle tracking systems and cosmic ray registering. To address this challenge, we devised a LPGA strategy that eliminates thermal and mass-transport instabilities, yielding perovskite films with exceptional vertical uniformity. This ensures depth-independent charge collection, thereby suppressing stochastic fluctuations in the final readout. With additional advantages of large-area processability and direct compatibility with readout circuits, our detectors deliver high-quality X-ray imaging at an effective per-pixel integration dose of 40.6 nGy_air_. If the technique described in this paper is combined with globally integrating readout synchronized with pulsed X-ray exposure, it holds the potential to translate into a proportional reduction in patient dose in the future. More broadly, together with recent progress in intelligent and dynamic perovskite X-ray imagers [47,48], such ultralow-dose imaging provides a sensor-level foundation for scalable, AI-enabled diagnosis, early screening, and population-level examinations.

Beyond X-ray detection, the demonstrated control of vertical homogeneity addresses a persistent bottleneck across perovskite optoelectronics. While uniformity is a decisive factor limiting the efficiency and stability of photovoltaics, light-emitting diodes, and other perovskite devices, our strategy renders vertical film homogenization comparatively straightforward even at much greater thicknesses. We anticipate this advance will not only redefine X-ray imaging but also establish a broadly applicable processing paradigm with lasting impact across diverse perovskite-based technologies.

## Supplementary Material

nwag391_Supplemental_File
