# Supplementary material for: Ultralow-dose X-ray imaging enabled by vertically homogeneous perovskite films
Source: Natl Sci Rev. 2026 Jun 25;13(15):nwag391. doi: 10.1093/nsr/nwag391 (PMC13455612; doi:10.1093/nsr/nwag391)
Supplement: Supplementary file 1 [file nwag391_supplemental_file.pdf]

Supporting Information

Methods ... 2
Note S1. Calculation of X-ray photon flux ... 6
Note S2. Poisson-governed depth-wise photon statistics and readout noise ... 6
Note S3. Simulation of the thermal field in traditional annealing and LPGA process ... 8
Note S4. Origin-domain decoupling of vertical and lateral non-uniformity ... 9
Note S5. Derivation of charge collection efficiency and detection efficiency ... 11
Note S6. Characterization of single-pixel detector noise ... 12
Note S7. Characterization of PRNU, DSNU and noise decomposition ... 13
Note S8. Characterization of DQE ... 14
Note S9. Characterization of modulation transfer function ... 15
Figure S1. The image noise for ideal film with perfectly uniform vertical transport ... 17
Figure S2. Monte Carlo simulations for film with nonuniform vertical transport ... 17
Figure S3. The experimental image noise ... 18
Figure S4. Schematic illustration of liquid-phase growth and annealing ... 18
Figure S5. Compatibility of anisole with typical perovskite precursor solvents ... 19
Figure S6. Material characterization of films from different annealing processes ... 19
Figure S7. Schematic diagram comparing liquid phase annealing and hot stage annealing ... 19
Figure S8. XPS characterization of films ... 20
Figure S9. Thermally stimulated current curves ... 20
Figure S10. Photoluminescence and TRPL characterization ... 21
Figure S11. Characterization of the X-ray absorption properties ... 22
Figure S12. Calculated detection efficiency and charge collection efficiency ... 22
Figure S13. Time-of-flight measurements ... 22
Figure S14. Characterization of X-ray response ... 23
Figure S15. Dark current-voltage characteristics ... 23
Figure S16. Temperature-dependent conductivity and Arrhenius plots ... 24
Figure S17. X-ray temporal response ... 24
Figure S18. The spatial response across eight device regions ... 24
Figure S19. Noise current spectra of LPGA and control devices under zero dose rate ... 24
Figure S20. Optical photograph of large-area perovskite thick films ... 25
Figure S21. Dark current density variation ... 25
Figure S22. Optical image of the TFT chip ... 26
Figure S23. PRNU, DSNU, and stacked-frame decomposition of temporal and spatial noise ... 27
Figure S24. The derived MTF and NPS curves ... 28
Table S1. The radiation quality of X-ray sources and corresponding parameters ... 28
Table S2. Material properties used for simulation ... 28
Table S3. Performance comparison for benchmark X-ray flat panel detectors ... 28
Table S4. Standard tissue weighting factors ... 29

Methods

Materials and reagents

Lead iodide (PbI_2, 99.999%), formamidinium iodide (FAI, 99.9%) and methylammonium iodide (MAI, 99.9%) were purchased from Advanced Election Technology Co., Ltd. N, N-dimethyl-1, 3-propylenediamine hydroiodate ($DMePDAI_2$) was purchased from Xi'an Yuri Solar Co., Ltd. Polyvinylpyrrolidone (PVP, Molecular weight 1,300,000), N,N-dimethylformamide (DMF, 99%), dimethyl sulfoxide (DMSO, 99%), gamma-butyrolactone (GBL, 99%), chlorobenzene (CB, 99%) were purchased from Aladdin.

Device fabrication

Firstly, polycrystalline perovskite seed layers were prepared by dissolving 0.1 g PVP, 0.75 mmol of FAI, 0.75 mmol of MAI, and 1.5 mmol of PbI_2 in a mixed solvent of DMF and DMSO (4:1 v/v, total volume 1 mL). The precursor was filtered through a 0.22 μm PTFE filter. Seed layers were deposited onto ITO substrates via a two-step spin-coating process: 500 rpm (acceleration 500 rpm s^{-1}, 10 s) and 3000 rpm (acceleration 2500 rpm s^{-1}, 30 s). During the second step, 300 μL of chlorobenzene was dropped. The coated film was transferred into preheated anisole (100°C) for annealing for 10 minutes.

For thick-film growth, FAI (150 mmol), MAI (150 mmol), and PbI_2 (300 mmol) were dissolved in 200 mL GBL with 5 wt% $DMePDAI_2$, and stirred at 60°C until clear. The solution was filtered (0.22 μm PTFE) into a 500 mL crystallization dish and heated at 90°C. As the solvent evaporated and perovskite nucleation commenced, the preheated seed layer was gently immersed in the crystallization dish for in-solution growth for 2 h, then removed. For annealing, anisole in a crystallization dish was preheated to 100°C and maintained for 20 min, after which the thick film was immersed for 30 min. The film was then taken out, and the residual solvent was removed by N_2 blowing to obtain the LPGA sample. For the control sample, the as-grown thick film was transferred onto a hotplate and annealed at 100°C for 8 h. Finally, a 50 nm Au electrode was thermally evaporated through a shadow mask to

form a glass/ITO/perovskite/Au structure with an active area of 0.0706 cm^2.

Materials characterization

Microscopic infrared spectra were measured using a microscope infrared spectrometer (Nicolet In10). TRPL mapping images were obtained using a laser confocal fluorescence lifetime imaging microscope (FLIM300), with a 405 nm excitation wavelength. SEM characterization was performed using a Hitachi SU8220 field emission scanning electron microscope. XRD patterns were recorded using a Cu Kα radiation X-ray diffractometer at a scan rate of 10°/min. Photoluminescence spectra were collected with a laser confocal Raman spectrometer (LabRAM HR800), with an excitation wavelength of 532 nm.

Trap-state and ionic transport characterization. Temperature-dependent measurements were performed using a liquid-nitrogen cryostat system with Au/perovskite/ITO devices (from top to bottom). For TSC measurements, the device was cooled to 88 K, illuminated with X-rays under RQA3 at 28.32 $\mu Gy_{air}\ s^{-1}$ for 10 min, and then heated in the dark from 88 to 225 K at 1 K per 15 s while the current was recorded. A baseline measured under the same thermal protocol without X-ray pre-illumination was subtracted to obtain the final TSC spectrum. For the temperature-dependent conductivity measurement, dark current-voltage curves were recorded from 140 to 320 K. The conductivity was extracted from the near-linear low-bias region and fitted using

$$\sigma(T) = \left(\frac{\sigma_0}{T}\right) \exp\left(\frac{-E_a}{K_B T}\right)$$

from which the apparent activation energy associated with ionic migration was obtained. Where $\sigma(T)$ is the conductivity at temperature T, σ_0 is a constant, E_a is the ion migration activation energy (eV), k_B is the Boltzmann constant (k_B = 8.617 × 10^{-5} eV K^{-1}), and T is the temperature (K).

Carrier transport characterization

μ and $\mu\tau$ were measured using Au/perovskite/ITO device structures (from top to bottom). A 365 nm LED was used as the excitation source and illuminated the device from the Au-electrode side. Dark-state and illuminated current-voltage curves were

recorded using a semiconductor parameter analyzer (FS-Pro) over the voltage range of 0–150 V, with positive bias applied to ITO and negative bias applied to Au. The net photocurrent-voltage curve was obtained by subtracting the dark current from the illuminated current, and the resulting net photocurrent was fitted using a modified Hecht equation to extract the $\mu\tau$ value. Under this measurement geometry, the fitted $\mu\tau$ is mainly governed by electron collection through the thick perovskite layer and is therefore regarded as an electron-transport-dominated effective $\mu\tau$.

μ was measured using a FlyToF system. A pulsed nitrogen laser (NL100, VSL-337i, λ = 337 nm) was used as the excitation source to generate transient photocurrent signals. The electron and hole mobilities were extracted by analyzing the corresponding carrier transit times in the transient photocurrent curves under an applied electric field, according to the relation $\mu = d^2/(\tau V)$, where d is the thickness of the perovskite layer, V is the applied bias, and τ is the carrier transit time.

X-ray detection and imaging

X-ray detection characterization was conducted using a medical X-ray tube (Leo, Varex imaging) operated at 50 kV. The X-ray spectrum was adjusted with a 0.3 mm Cu plate and a 1 mm Al plate to meet the RQA3 standard. The tube current was tunable from 25 to 200 mA. Device X-ray responses were recorded using a semiconductor parameter analyzer (PRIMARIUS, FS-Pro). For imaging, the TFT readout system (LinkZill Technology Co., Ltd) comprised a 64 × 64 pixel array, with an active area of 12.8 mm × 12.8 mm and a pixel pitch of 200 μm. The readout integrated circuit model Zhihe 03MR provided 64 current readout channels with a detection range of 100 pA–200 nA. The acquired signals were displayed as 8-bit images. For imaging over extended fields of view, a high-precision XY-axis motorized stage (minimum step size: 10 μm) was used for sequential image stitching. Multiple sub-images were captured by translating the object across the imager's active area, followed by post-acquisition alignment and merging. Specifically, stitched images were generated from 1 × 2 frames for the molar tooth, 11 × 5 frames for the bat specimen, 3 × 3 frames for the walnut slice, and 5 × 5 frames for the orange slice.

During imaging, the prototype operated in a row-by-row current-mode readout mode, in which photogenerated charge was stored in external capacitors integrated in the ADC rather than in in-pixel storage capacitors. The X-ray source remained on during the acquisition of one full frame. Therefore, each pixel integrated signal only during its row activation time, whereas the sample was continuously irradiated throughout the full-frame scan. Under a frame rate of 10.88 fps and 64-row sequential readout, the row activation time was 1.436 ms.

Note S1. Calculation of X-ray photon flux.

The incident X-ray photon count per pixel is calculated as follows:

$$\varphi = \mathrm{SNR}_{\mathrm{in}}^2 \times D \times A \quad (1)$$

where SNR_{in}^2 represents the squared signal-to-noise ratio per unit dose and unit area under a specific radiation quality. Radiation quality is defined according to IEC 61267:1994 by the half-value layer and specified additional filtration (**Table S1**). D denotes the absorbed dose in μGy_{air}, and A is the photosensitive area (0.01 in mm^2).

In clinical digital radiography systems, the commonly used radiation quality is RQA5, with typical single-exposure doses of 1.25 μGy_{air}. Under these conditions, the incident photon count per pixel is estimated to be ~377. In this study, all X-ray imaging measurements were performed under RQA3 radiation quality, with effective per-pixel integration doses ranging from 25.9 to 80.1 nGy_{air} under the present row-selective readout architecture.

Note S2. Poisson-governed depth-wise photon statistics and readout noise under depth-dependent charge collection efficiency.

X-ray photons arrive at the detector surface randomly in both space and time, and therefore the photon count n follows a Poisson distribution, with mean E(n)=λ and variance Var(n) = λ. During subsequent propagation inside the detector, photon absorption in a thin layer of thickness dt is a stochastic thinning process, in which each photon is absorbed with probability p. According to the Poisson-thinning theorem, the number of photons absorbed in any depth interval (0, t_0) remains Poisson-distributed, with the corresponding mean and variance evolving with depth. Importantly, this depth-wise fluctuation in photon deposition is an intrinsic consequence of sparse-photon absorption statistics and exists independently of any particular transport or collection parameter of the detector itself.

For the 500 μm-thick $FA_{0.5}MA_{0.5}PbI_3$ film studied here, if the absorber is divided into two 250 μm layers, the Beer–Lambert law predicts that, on average, ~8 photons are absorbed in the upper layer and ~2 photons in the lower layer when 10 photons are

incident. However, because absorption is governed by Poisson/binomial statistics, individual pixels can deviate substantially from this average distribution, for example showing 9 and 0–1 photons or 7 and 2–3 photons in the two layers, respectively. Consequently, the actual depth profile of absorbed photons is not perfectly smooth, but fluctuates statistically from pixel to pixel, and this fluctuation becomes increasingly pronounced at low photon flux. Whether such intrinsic absorption-depth fluctuations remain statistically averaged out or are further converted into excess output noise depends on the subsequent depth-dependent weighting of signal generation, transport, recombination, gain, and collection, as discussed below.

For the final readout signal, incident photons are converted into charge carriers by the semiconductor layer. Variations in film density, grain size, crystallinity, or electrical gain across successive dt slices of the detector result in layer-dependent charge-collection efficiencies (CCE) and gain. To clarify the physical origin of the noise amplification, we discretize the absorber thickness into multiple layers, and the number of photons absorbed in layer (depth) k is $n_k \sim$ Poisson (λ_k), the total collected signal can be written as:

$$S = q \sum_k \eta_k n_k$$

where q is the collected charge generated per fully collected event, and η_k is the effective layer-dependent collection/gain factor. Then,

$$E[S] = q \sum_k \eta_k \lambda_k$$

and

$$\mathrm{Var}(S) = q^2 \sum_k \eta_k^2 \lambda_k$$

Defining the absorption-weighted average collection factor as

$$\bar{\eta} = \frac{\sum_k \eta_k \lambda_k}{\sum_k \lambda_k}$$

and the total absorbed photon number as $N_{abs} = \sum_k \lambda_k$, the variance can be rewritten as

$$\mathrm{Var}(S) = q^2\bar{\eta}^2 N_{abs} + q^2 \sum_k \lambda_k \left(\eta_k - \bar{\eta}\right)^2$$

The first term is the Poisson-limited contribution expected even for a vertically uniform absorber. The second term is an excess variance term arising exclusively from the depth dependence of η_k. Therefore, when the film is vertically homogeneous ($\eta_k=\bar{\eta}$), the excess term vanishes and fluctuations in photon absorption depth do not lead to additional noise amplification. In contrast, when η_k varies with depth, the stochastic absorption profile is weighted unequally, which directly amplifies the output noise.

From this perspective, trap-assisted recombination is not excluded from the mechanism; rather, it enters the model through its impact on the local collection factor ηk. More specifically, in this framework, ηk should be understood as an effective layer-dependent collection/gain "sum/macro-level" factor. It incorporates multiple microscopic contributions, including local charge-generation yield, CCE, trap-assisted recombination loss, photoconductive/electronic gain, and possible local-field effects. These quantities are not independent additive noise sources in the depth-weighting model; instead, these depth-dependent signal weights then convert stochastic absorption-depth variations into the observed excess output variance.

Here, we assume that the gain within the total detector thickness 0–t_0 follows a Gaussian distribution with mean G and variance σ_{IRN}^2. Monte Carlo simulations were performed with 10,000 events. The input parameters included the mean photon count λ, the per-layer absorption probability p, and CCE. The resulting signal-to-noise ratio (SNR) was then calculated. **Figures S2** show SNR for p = 2%, 5%, 10%, 20%, 30%, 40% and 50% (each layer = t_0/5), with the abscissa representing λ (10–1000 photons) and the normalized gain variance σ_{IRN}^2 (0–2.0), and the ordinate sharing a common SNR scale of 1–31.6. The results indicate that smaller λ and p yield lower SNR. In thick films, σ_{IRN}^2 is typically > 1, making the intrinsic readout noise comparable in magnitude to that of the photon-counting statistics.

Note S3. Simulation of the thermal field in traditional annealing and LPGA

process.

Finite element analysis was performed using SolidWorks to simulate heat transfer in the system. Models were created for both glass and perovskite, each with dimensions of 0.18×0.18×0.5 mm^3. Hexahedral elements were used for the heat transfer analysis, with the glass model containing 50,000 elements, and the perovskite model containing 100,000 elements. Material properties, including thermal conductivity, specific heat, and density, are listed in **Table S2**.

The initial temperature of all models was set to 25 °C. Heat transfer between the glass and perovskite contact surface was defined with a thermal conductivity coefficient of 1500 W/m^2/°C specified. For simplification, the hot plate, solvent medium, glass and perovskite were treated as isothermal bodies. The initial surface temperature of the perovskite immersed in the solvent was set to 100 °C, with a surface film coefficient of 0.5 W/m^2/°C, while the perovskite surface exposed to gas was set to 25 °C with a surface film coefficient of 0.28 W/m^2/°C. The heat transfer process was simulated using transient analysis, with a total simulation time of 20 s and a time step of 0.01 s.

Note S4. Origin-domain decoupling of vertical and lateral non-uniformity.

To clarify why vertical uniformity plays a particularly important role in thick direct-conversion X-ray detectors, it is useful to distinguish between the physical origin of noise inside a three-dimensional detector pixel and the output manifestation of noise in a two-dimensional image. In this framework, each pixel should be regarded not as a purely two-dimensional sensing element, but as a three-dimensional volumetric detector unit.

In this device-domain description, the pixel response can be written schematically as Noise = Noise (x, y, z). Non-uniformity along the film-thickness direction z contributes to a vertical-origin noise component, including through-thickness variation in crystallinity, defect density, carrier lifetime, local electric field, CCE, and effective gain. By contrast, non-uniformity within the x-y plane contributes

to a lateral-origin noise component, including in-plane variation in morphology, local contact, pixel interface, and regional photoresponse. Importantly, both components may contain quasi-static and stochastic/time-dependent parts, and therefore can contribute to both image-domain spatial noise and temporal random noise.

When moving from a single three-dimensional detector pixel to a two-dimensional imaging array, the measured image no longer contains an explicit z-axis. Each image pixel provides only one output value, which is the integrated result of all X-ray absorption, charge generation, transport, recombination, gain, and collection processes occurring inside that three-dimensional detector volume. Therefore, image-domain spatial noise—such as fixed-pattern noise (FPN), photo-response non-uniformity (PRNU), or dark-signal non-uniformity (DSNU)—is an output metric, not a unique physical origin. It may arise from lateral-origin non-uniformity, vertical-origin non-uniformity, or both.

This distinction is especially important because, in many conventional thin-film visible-light image sensors, the active layer is sufficiently thin that the device response can often be approximated as quasi-two-dimensional, i.e., Noise(x, y, z) ≈ Noise(x, y). Under that approximation, image-domain spatial noise is naturally associated with lateral pixel-to-pixel variation. However, this simplification is no longer valid for hundred-micrometer-thick perovskite X-ray detectors. In such devices, X-ray photons are absorbed stochastically throughout a thick three-dimensional absorber, and the carrier drift distance is comparable to the film thickness. If CCE, carrier lifetime, trap-assisted recombination loss, or effective gain varies along the thickness direction, photons absorbed at different depths contribute unequally to the final pixel output. Under low-dose conditions, where only a small number of photons are absorbed per pixel, stochastic absorption-depth fluctuations are therefore converted into excess output variance. This is the vertical-origin noise mechanism emphasized in our work.

Consequently, through-thickness non-uniformity in CCE/gain can still manifest as PRNU or FPN in the final two-dimensional image, because different pixels may

possess different depth-dependent response profiles. Thus, PRNU is a horizontal image-domain output metric, but not necessarily a purely lateral-origin noise source. The reduction of PRNU after LPGA treatment therefore does not contradict our vertical-uniformity mechanism; rather, it indicates that homogenizing the through-thickness response of the perovskite thick film makes the volume-integrated output of different pixels more consistent. In this sense, LPGA suppresses a vertical-origin material/device non-uniformity, which is ultimately observed as reduced image-domain spatial noise and improved temporal stability in the imaging array.

To further support this interpretation, we additionally performed cross-sectional TRPL mapping over a wider lateral field of view, which enabled a direct comparison of lateral and vertical variations within the same cross-sectional measurement window. The results show that the control thick film is relatively uniform laterally over the measured range, but exhibits pronounced through-thickness non-uniformity, whereas the LPGA thick film is uniform in both the lateral and vertical directions within the same measurement window (**Figure S10c and Figure S10d**). Quantitatively, the standard deviation of the TRPL lifetime in the selected lateral region is only 2.51 ns for the LPGA film (region A) and 6.26 ns for the control film (region A'), while in the selected vertical region it is 3.79 ns for the LPGA film (region B) and 12.54 ns for the control film (region B'). Thus, in the control film, the vertical variation is substantially larger than the lateral variation, whereas in the LPGA film both variations remain small. This comparison highlights the particular importance of vertical uniformity: although lateral uniformity is also necessary for high-quality imaging, the present results indicate that the key difference between the control and LPGA films lies in the suppression of through-thickness non-uniformity, which therefore plays the dominant role in reducing noise amplification in this thick X-ray absorber. Consistently, this improvement is ultimately manifested at the imaging level by a reduction of the PRNU from 8.05% to 0.68% and the DSNU from 5401.09 e^- to 627.27 e^-.

Note S5. Derivation of charge collection efficiency and detection efficiency.

The charge collection efficiency (CCE) and detection efficiency (DE) were calculated as follows (*Nat. Photon.* **18**, 1052-1058 (2024)):

$$\mathrm{CCE} = \frac{\mu\tau E}{d} \times \left(1 - \exp\left(-\frac{d}{\mu\tau E}\right)\right)$$

$$\mathrm{DE} = \left(1 - \mathrm{e}^{-\alpha d}\right) \times \mathrm{CCE}$$

where $\mu\tau E$ is the Schubweg distance, d is the thickness of the absorption layer and α is the linear attenuation coefficient. In detail, X-ray tube voltage of 50 kVp was used, and the peak energy of 42.6 keV was taken as representative of the mean photon energy. Under these conditions, the linear attenuation coefficient of the perovskite layer is 75 cm^{-1}.

Note S6. Characterization of single-pixel detector noise.

The Noise Equivalent Dose (NED) was measured using a spectrum analyzer (Agilent 35670A). The test system used a shielding enclosure or shielded cables to reduce electromagnetic interference from the environment. Also, the test system should be grounded to avoid ground loop noise and radio frequency interference. The external vibrations or mechanical noise should be minimized by placing the system on a stable optical platform. The bias voltage of the device was set at 5 V. The preamplifier SR570 was set with a gain 10^6 A/V. The spectrum analyzer was set to logarithmic mode, and the bandwidth is between 10 Hz–3,000 Hz. Subsequently, the noise was recorded at different dose rates of 0, 18, 61, 104, 147, 191, and 234 $\mathrm{nGy_{air}\ s^{-1}}$, respectively.

The total noise (N_T) of the detector as a function of X-ray dose rate is expressed in terms of the noise-current spectral density (*Nat. Photon.* **17**, 510-517 (2023)):

$$N_T^2 = PSN^2 + DN^2$$

where *PSN* denotes the photon shot noise, and *DN* represents the dark current noise.

$$PSN^2\left[\frac{\mathrm{A}^2}{\mathrm{Hz}}\right] = 2 \times \mathrm{DE} \times A^2 \times S_{th}^2 \times D_1 \times D_r$$

where *DE* is the detection efficiency, *A* is the area, S_{th}^2 is the theoretical sensitivity, D_1 represents the radiation dose corresponding to the flux of a single photon through area

A, and D_r is the dose rate.

The detector's NED can be expressed as:

$$\mathrm{NED}[\mathrm{Gy_{air}}] = \frac{DN^2\left[\frac{\mathrm{A}^2}{\mathrm{Hz}}\right]}{2 \times \mathrm{DE} \times A^2 \times S_{th}^2 \times D_1}$$

From this relation, the NED can be obtained by fitting the noise-current spectra measured at different dose rates. To ensure an accurate NED, an appropriate frequency range must be selected from the noise spectrum. At low frequencies, *1/f* noise increases the noise-current spectral density and thus the NED. At frequencies above the detector's f_{3dB}, the decline in DE also increases NED. Therefore, the optimal sampling frequency range lies between the *1/f* corner and f_{3dB}. For our detector, the *1/f* corner is ~60 Hz (**Figure S19**), and f_{3dB} is ~194 Hz (**Figure S17**). Based on these values, 160 Hz was selected as the operating bandwidth for NED measurements.

Note S7. Characterization of photo-response non-uniformity (PRNU), dark signal non-uniformity (DSNU), and noise decomposition for the array detector.

According to the European Machine Vision Association (EMVA) 1288 standard, PRNU and DSNU are defined as:

$$\mathrm{PRNU}_{1288} = \frac{\sqrt{s_{y.50}^2 - s_{y.\mathrm{dark}}^2}}{\mu_{y.50} - \mu_{y.\mathrm{dark}}}$$

$$\mathrm{DSNU}_{1288} = \frac{s_{y.\mathrm{dark}}}{K}$$

where $s_{y.\mathrm{dark}}^2$ and $s_{y.50}^2$ denote the spatial variances of the dark field and the 50% saturated images, respectively; $\mu_{y.50}$ and $\mu_{y.\mathrm{dark}}$ represent the corresponding mean gray levels; and K is the global system gain.

The image noise σ_{IN} can be expressed as:

$$\sigma_{IN} = s_{y\cdot 50} \times K'$$

where $s_{y.50}$ is the spatial standard deviation of the gray values for the 50% saturated image, and K' is the conversion factor from digital number (DN) to electron charge

(e^{-}/DN).

The conversion factor K' is calculated as:

$$K' = \frac{Q}{e \times 2^N} = \frac{CU}{e \times 2^N}$$

where Q is the full-well charge of the pixel, C is the pixel capacitance, U is the capacitor voltage, e is the elementary charge, and N is the ADC bit depth.

To further distinguish temporal random noise from spatial fixed-pattern noise, stacked-frame analysis was performed for both dark and illuminated images. Here, N=1 denotes a single acquired frame, whereas N>1 denotes the image obtained by averaging N consecutively acquired frames. Because the temporal variance decreases as 1/N upon frame averaging, while the fixed-pattern component remains unchanged, the measured variance can be decomposed into temporal and spatial contributions. For the dark state, the relationship can be written as:

$$\sigma_{dark}^2(N) = \frac{\sigma_{temp,dark}^2}{N} + DSNU^2$$

where $\sigma_{\text{temp,dark}}$ is the temporal random dark-noise component and the asymptotic value at large N corresponds to the DSNU. For the illuminated state, the corresponding relative non-uniformity follows:

$$\left(\frac{\sigma_{light}}{\mu_{light}}\right)^2(N) = \frac{\sigma_{temp,light}^2}{N} + PRNU^2$$

where $\sigma_{\text{temp,light}}$ denotes the temporal random component under illumination and the asymptotic value at large N corresponds to the PRNU. Therefore, the difference between the single-frame variance and the asymptotic variance provides a quantitative estimate of the temporal random-noise contribution.

Note S8. Characterization of detection quantum efficiency (DQE).

During x-ray imaging, charge storage is achieved using external capacitors integrated in the analog-to-digital converter (ADC). The TFT array operates with a row-by-row scanning and column-parallel readout scheme. Since each row collects photogenerated charge only during its activation period, the effective exposure time per pixel corresponds to the duration of row activation. Therefore, the effective per-

pixel integration dose is obtained by dividing the incident dose rate by the product of the frame rate and the number of rows. Under a dose rate of 28.32 μGy_{air} s^{-1}, a frame rate of 10.88 fps, and 64-row sequential readout, the row activation time is 1/(10.88×64) s = 1.436 ms, corresponding to an effective per-pixel integration dose of 40.6 nGy_{air}. Because the X-ray source remained on during the acquisition of one full frame in the present prototype, the total sample entrance dose per full-frame acquisition was 28.32/10.88 = 2.60 μGy_{air}. This pixel-level effective integration dose was used for DQE calculation because it determines the incident photon fluence associated with the signal formation of an individual pixel under the present row-selective readout architecture, whereas the total sample entrance dose per full-frame acquisition reflects system-level irradiation during the full-frame scan.

The detective quantum efficiency (DQE) as a function of spatial frequency f is evaluated according to the European Machine Vision Association standard:

$$\mathrm{DQE}(f) = \frac{\mathrm{SNR}_{\mathrm{out}}^2(f)}{\mathrm{SNR}_{\mathrm{in}}^2(f)} = \frac{q \times G^2 \times \mathrm{MTF}^2(f)}{\mathrm{NPS}(f)}$$

where q is the incident X-ray photon fluence per unit area, *G* is the system gain, MTF(*f*) is the modulation transfer function, and NPS (*f*) is the noise power spectrum.

For a single-pixel device, corresponding to the zero-frequency limit ($f \to 0$), the theoretical DQE can be expressed by the simplified model (*Nat. Photon.* **17**, 510-517 (2023)):

$$\mathrm{DQE} = \frac{\mathrm{DE}}{1 + \frac{\mathrm{NED}}{D}}$$

where DE is the intrinsic detection efficiency, NED is the noise-equivalent dose, and *D* is the incident dose.

Note S9. Characterization of modulation transfer function (MTF).

The MTF was evaluated using the slanted-edge method. A thin steel slice was placed under X-ray irradiation to acquire a one-dimensional edge profile. The edge spread function (*ESF(x)*) was extracted from this profile, and the line spread function (*LSF(x)*) was subsequently obtained by differentiating *ESF(x)*. The *MTF* was then

calculated as:

$$\mathrm{MTF}(v) = F\big(\mathrm{LSF}(x)\big) = F\left(\frac{d\mathrm{ESF}(x)}{dx}\right)$$

where v is the spatial frequency and x is the spatial position along the edge. MTF(v) is the Fourier transform (F) of LSF(x).

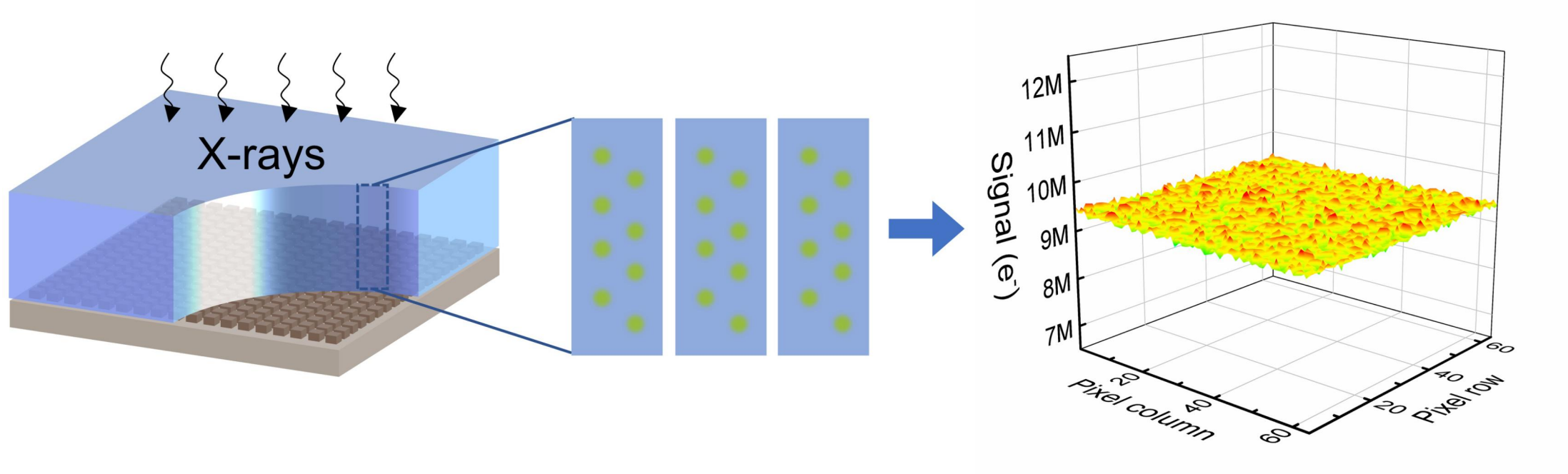

Figure S1. The image noise for ideal film with perfectly uniform vertical transport.

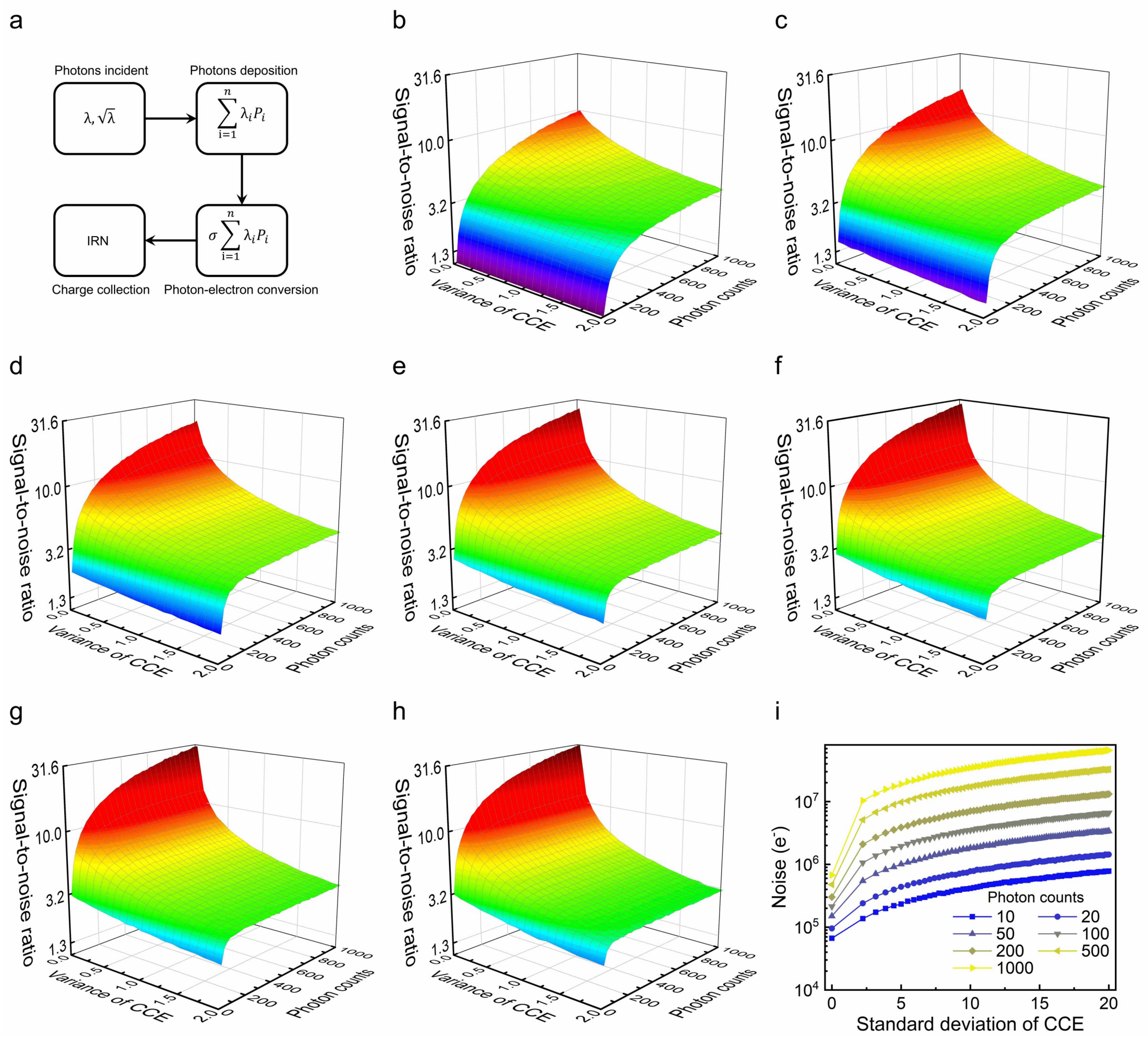

Figure S2. Monte Carlo simulations for film with nonuniform vertical transport. (a) The process of generating the output signal of the detector. The calculated signal-to-noise ratio with per-layer absorption probability p of 2% (b), 5% (c), 10% (d), 20% (e), 30% (f), 40% (g) and 50% (h). (i) Dependence of image noise with incident photon count and the standard deviation of layer-specific CCE under the condition of

p=0.5.

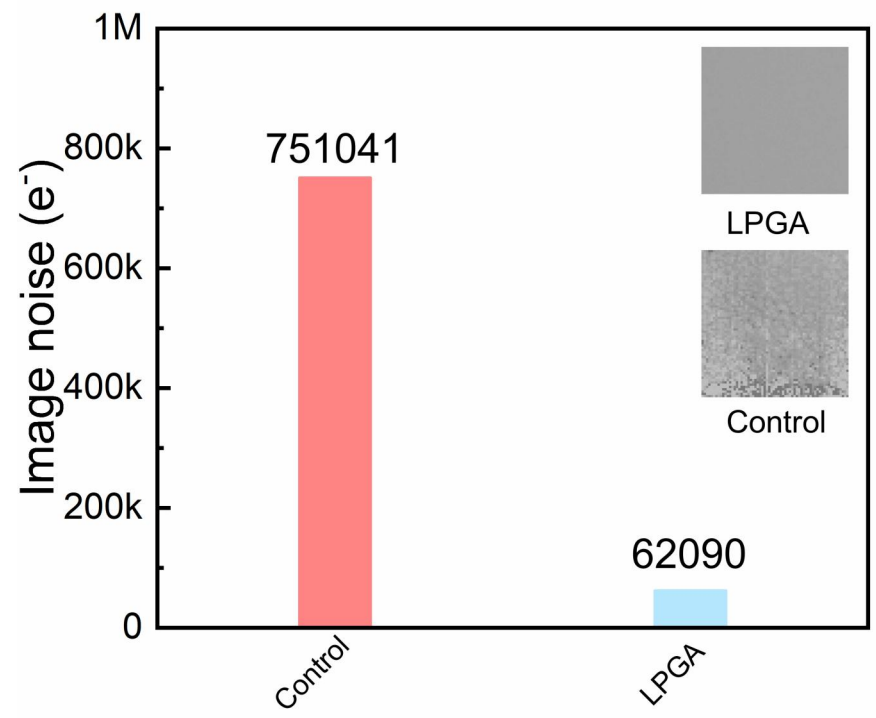

Figure S3. The experimental image noise. Inset is the image used to calculate the image noise.

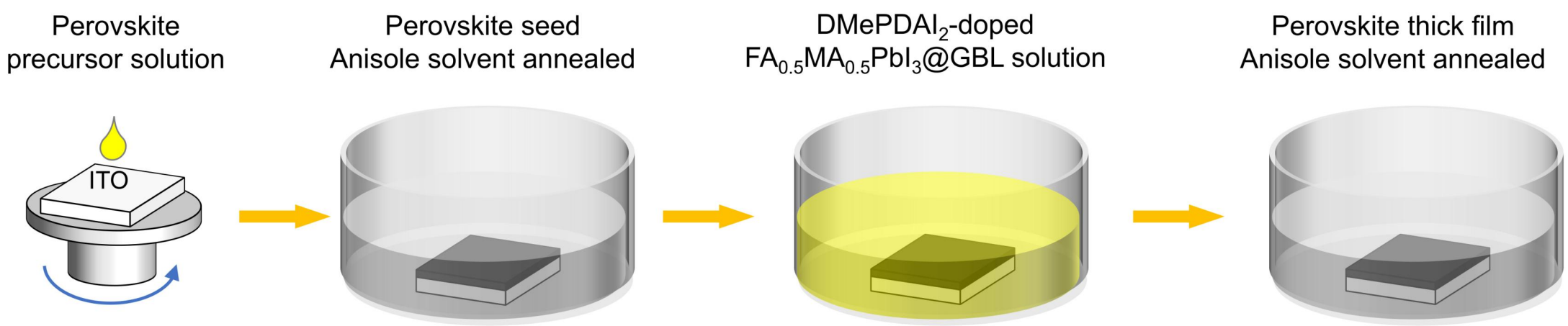

Figure S4. Schematic illustration of liquid-phase growth and annealing. The preparation of perovskite thick films via liquid-phase growth and annealing. A polyethylene pyrrolidone (PVP)-perovskite composite polycrystalline film was first employed as a growth template, where PVP mitigates thermal stress between the perovskite layer and the substrate, thereby improving interfacial contact. Subsequently, $DMePDAI_2$-doped $FA_{0.5}MA_{0.5}PbI_3$ thick films were deposited using an in situ chemical bath deposition (CBD) method. The incorporation of a small amount of $DMePDAI_2$ slows the crystal growth rate, leading to improved crystallinity and overall film quality. Finally, the as-deposited perovskite thick film was subjected to liquid-phase annealing.

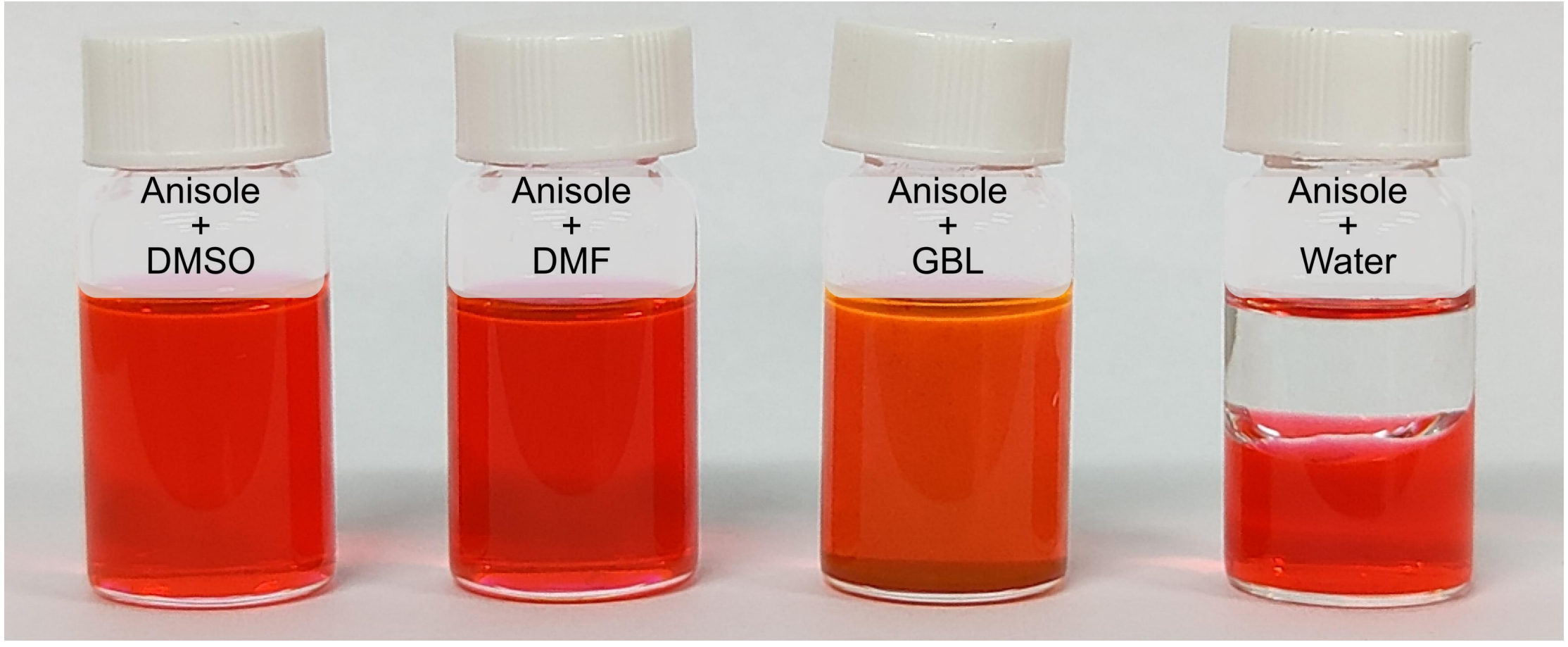

Figure S5. Compatibility of anisole with typical perovskite precursor solvents. From left to right: anisole (1 mL) + DMSO (1 mL), anisole (1 mL) + DMF (1 mL), anisole (1 mL) + GBL (1 mL), and anisole (1 mL) + water (1 mL). A small amount of red ink was added to DMF, DMSO, GBL, and water to facilitate visualization.

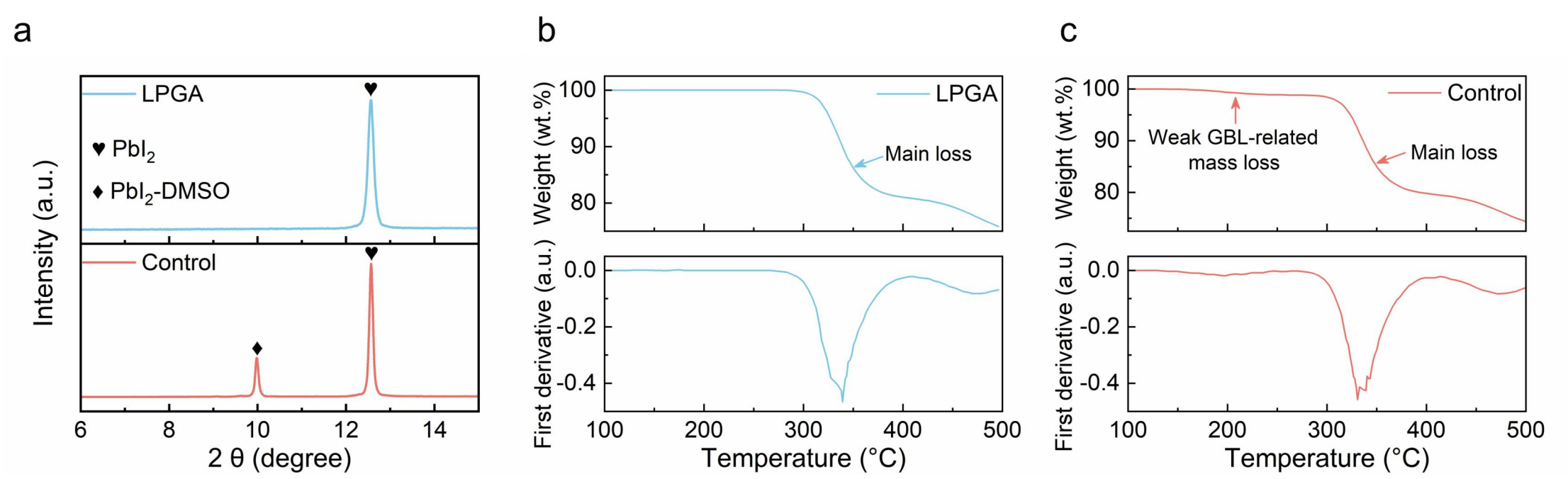

Figure S6. Material characterization of films from different annealing processes. (a) XRD patterns of PbI_2/DMSO after 30 seconds of annealing at 100 °C under both hotplate annealing and anisole medium. TGA curve of the (b) LPGA and (c) Control perovskite thick film.

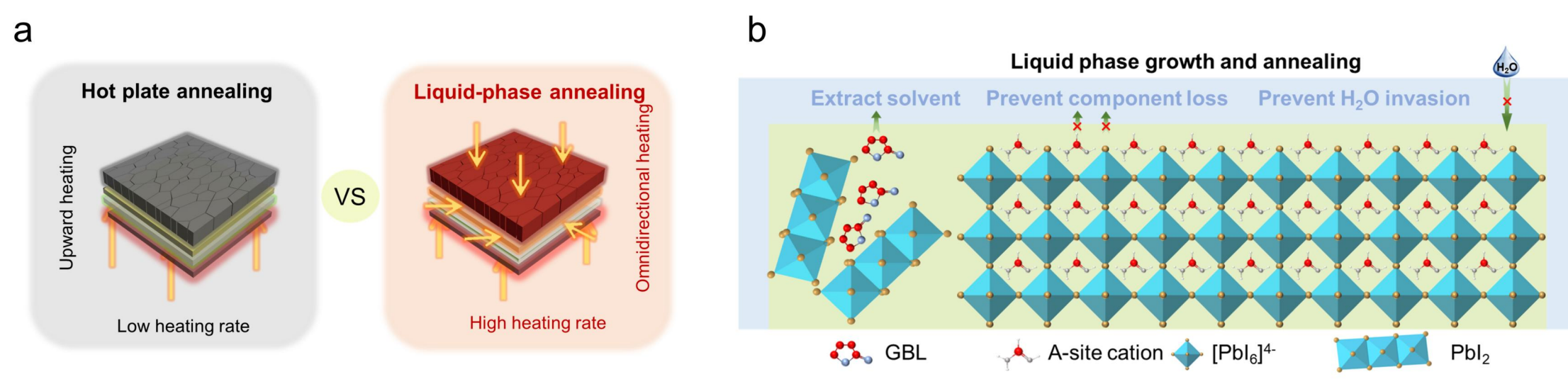

Figure S7. Schematic diagram comparing liquid phase annealing and hot stage annealing (a) Schematic diagrams of hotplate annealing and liquid-phase annealing.

(b) Advantages of LPGA methods.

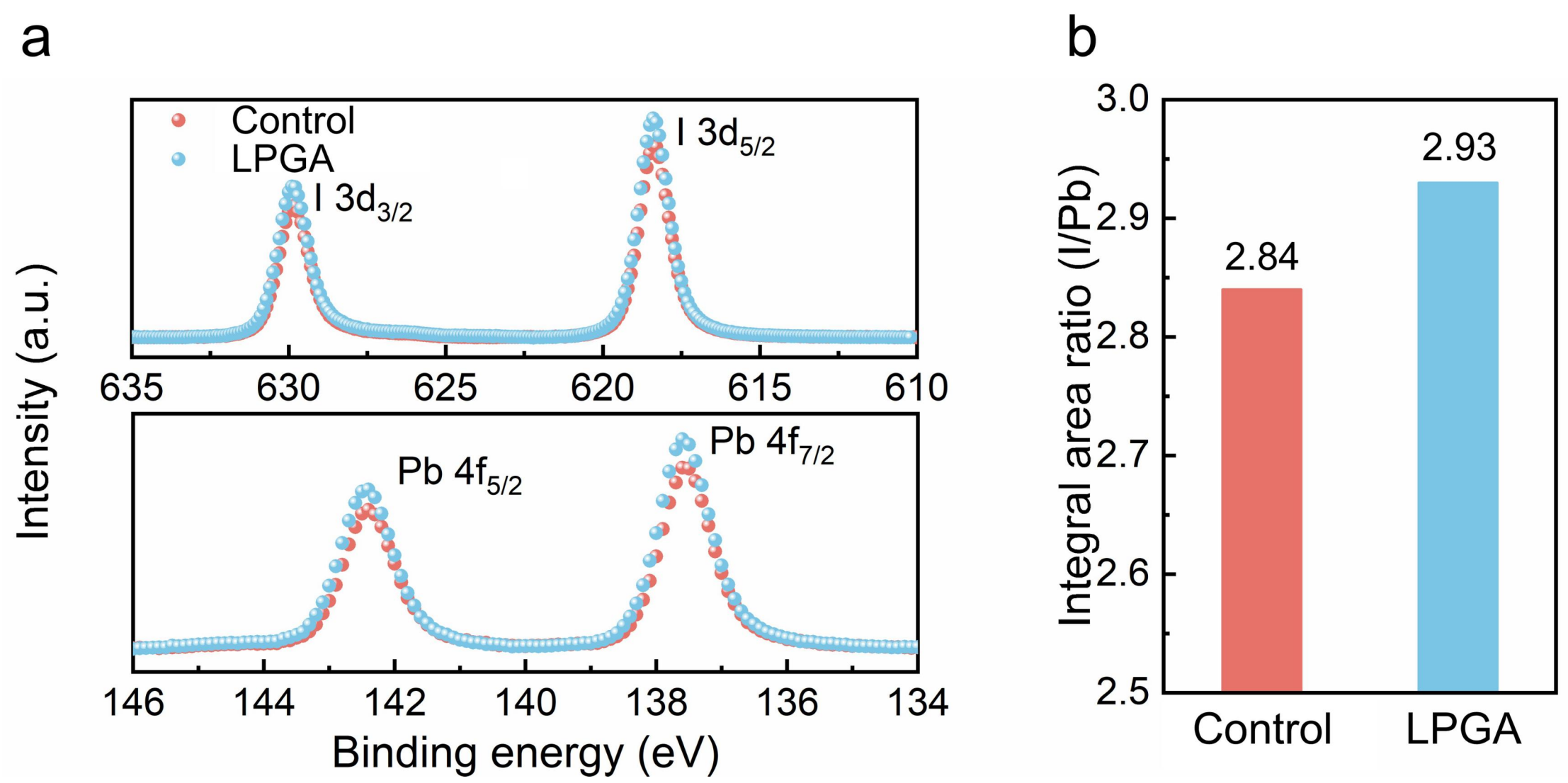

Figure S8. XPS characterization of films from different annealing processes. (a) XPS fine spectra of I_{3d} and Pb_{4f} signals for perovskite films. (b) Calculated I/Pb ratio obtained from the integral areas of the XPS peaks.

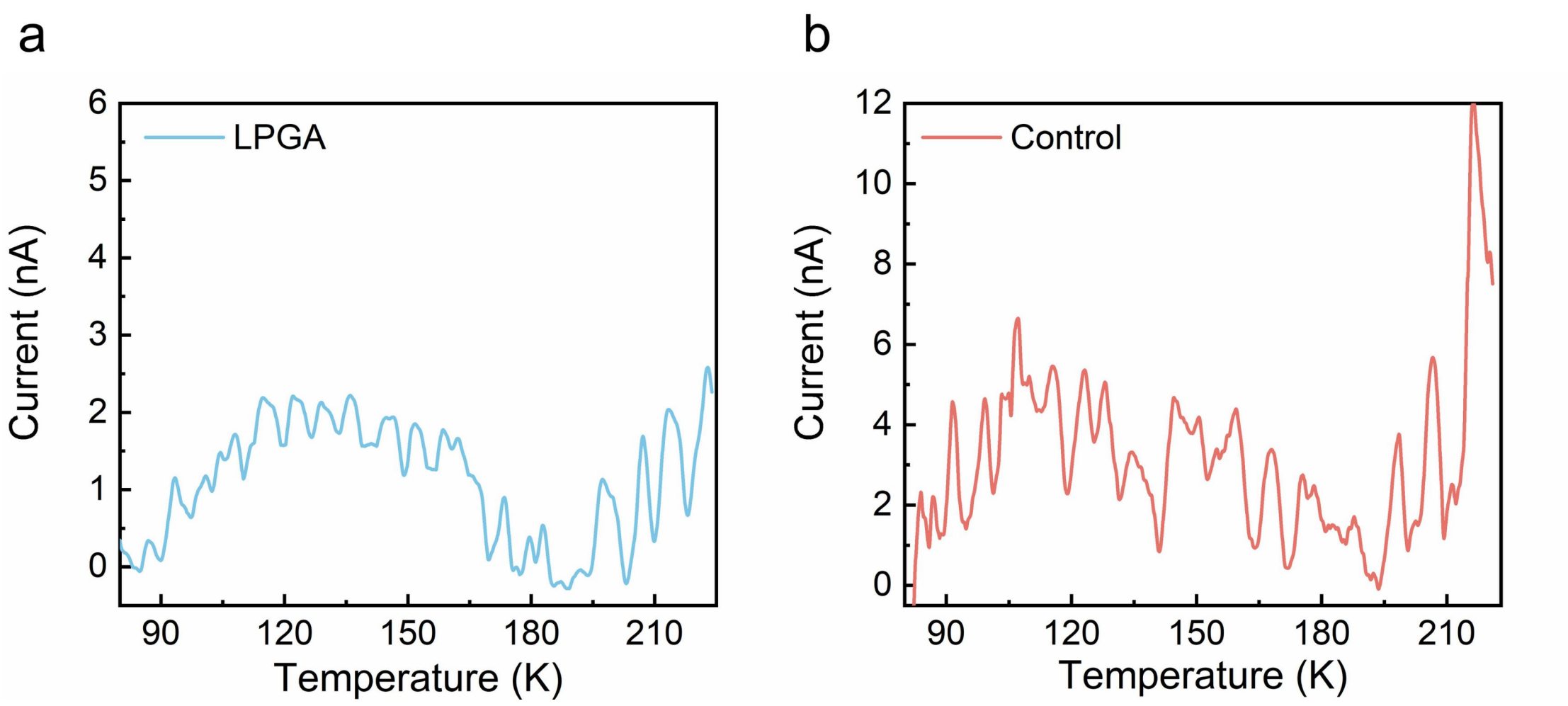

Figure S9. Thermally stimulated current (TSC) curves of the control and LPGA thick films. (a) LPGA device. (b) control device.

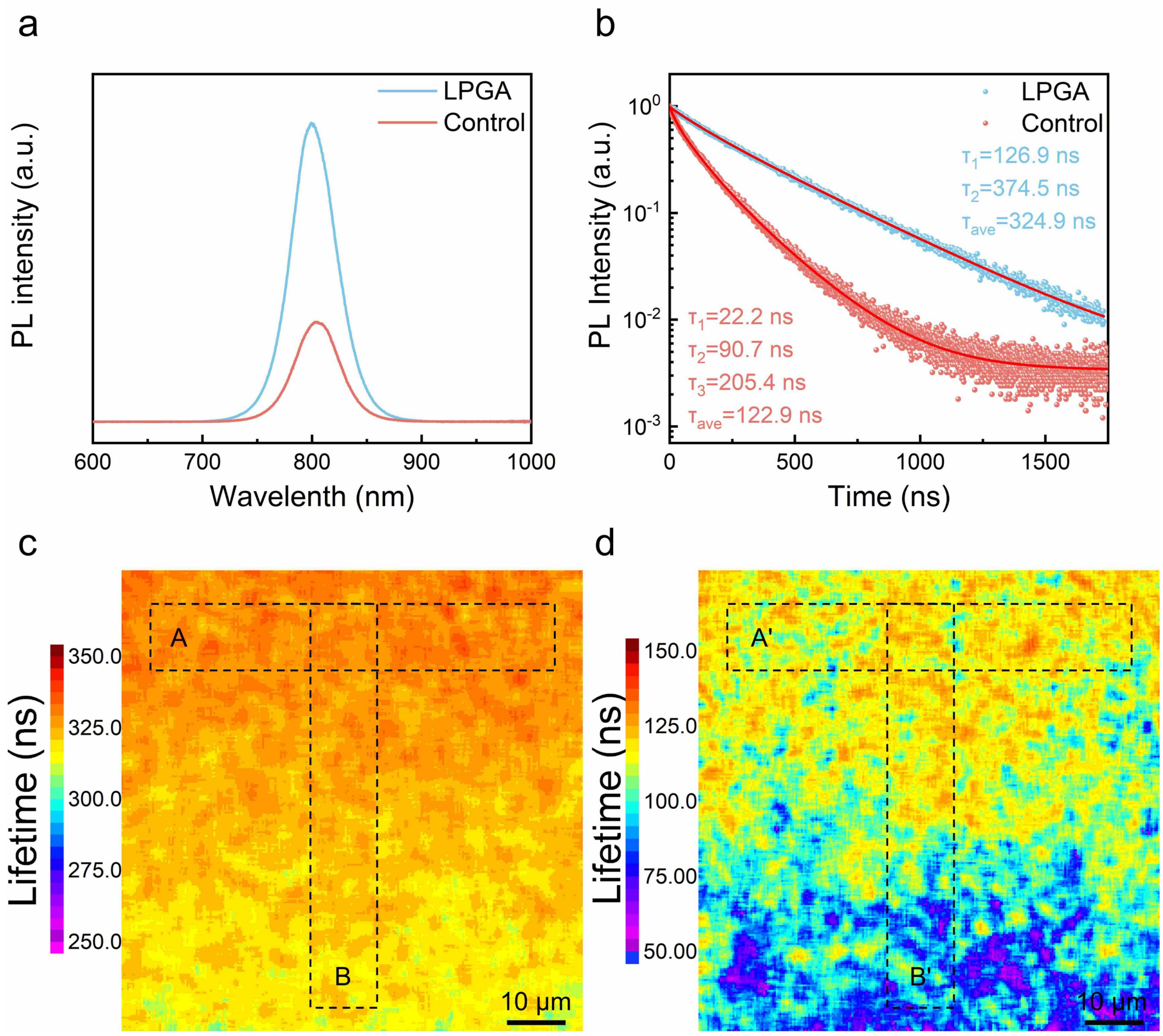

Figure S10. Photoluminescence and TRPL characterization of the perovskite films. (a) PL spectra and (b) TRPL curves of the control and LPGA samples. (c,d) Wide-field cross-sectional TRPL lifetime maps of the LPGA and control thick films, respectively. The selected lateral regions (A and A') and vertical regions (B and B') were used to compare lateral and through-thickness lifetime variations within the same cross-sectional measurement window. The standard deviations of the lifetime are 2.51 ns (A) and 3.79 ns (B) for the LPGA film, and 6.26 ns (A') and 12.54 ns (B') for the control film, indicating that the control film is relatively uniform laterally over the measured range but exhibits much stronger through-thickness non-uniformity, whereas the LPGA film remains uniform in both directions.

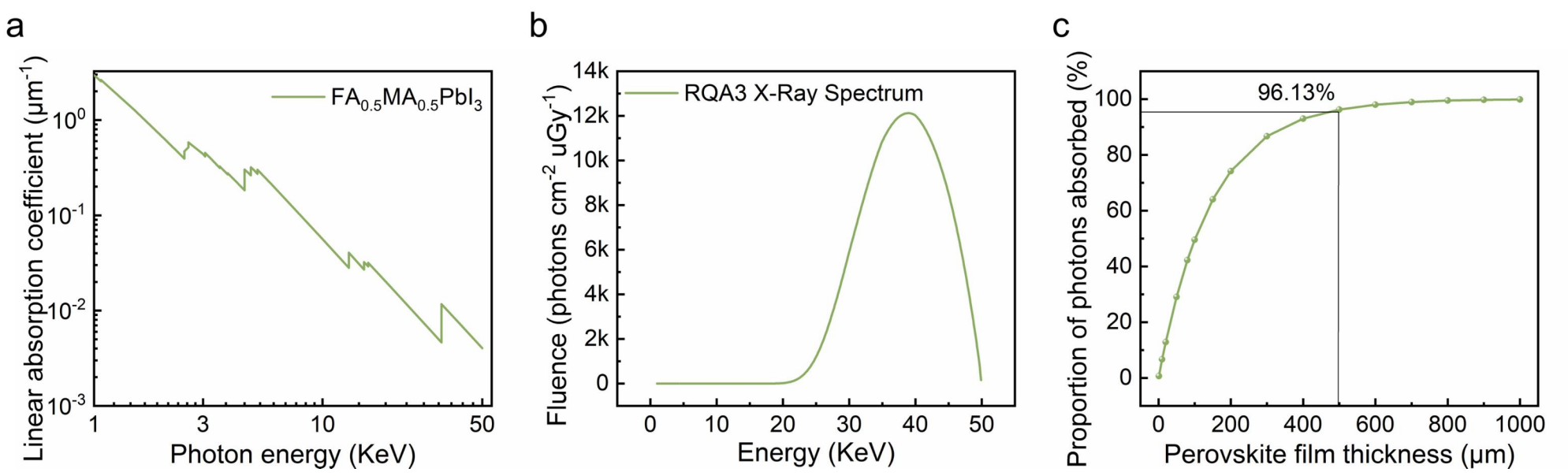

Figure S11. Characterization of the X-ray absorption properties for the perovskite films. (a) Linear absorption coefficients of perovskite at different X-ray energies. (b) Photon flux density of the X-ray spectrum for RQA3. (c) Dependence of photon absorption ratio on the film thickness.

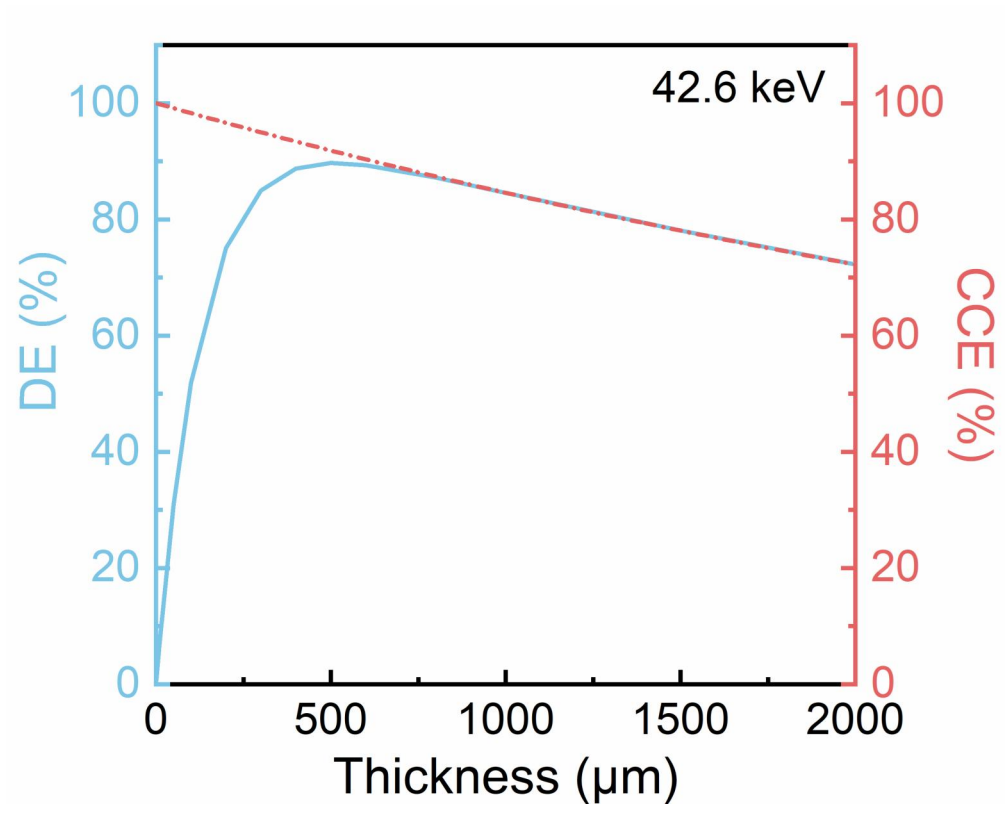

Figure S12. Calculated detection efficiency and charge collection efficiency for perovskite films.

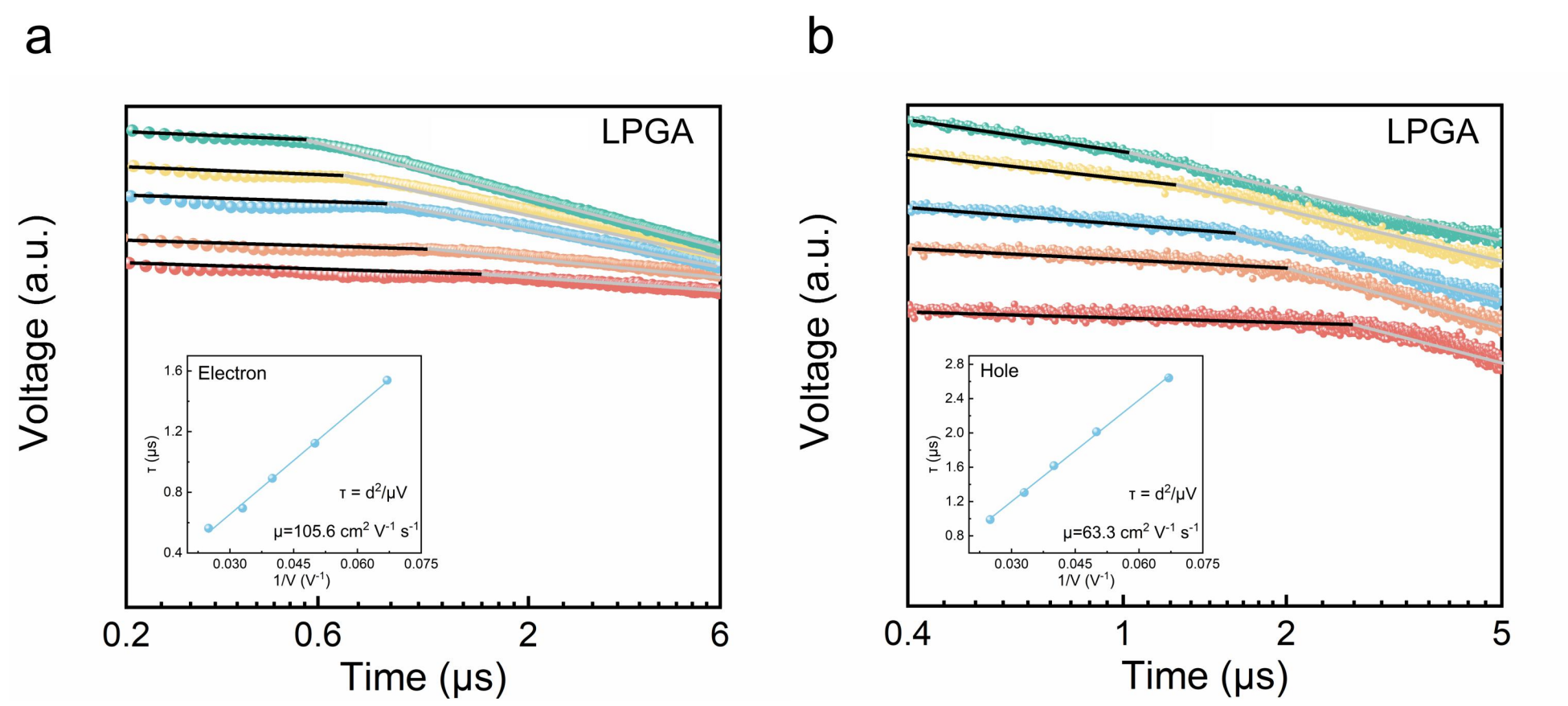

Figure S13. Time-of-flight measurements for the LPGA-processed films. (a) Electron mobility and (b) hole mobility.

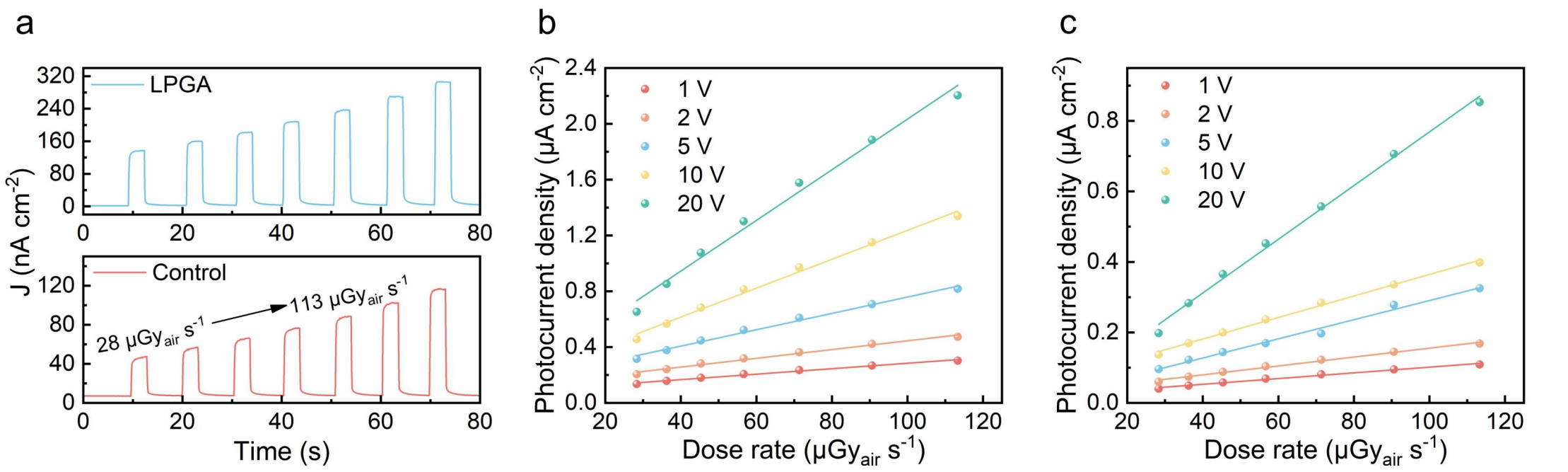

Figure S14. Characterization of X-ray response. (a) X-ray response of the control and LPGA devices under different dose rates at a bias of 1 V. Dose-rate-dependent photocurrent of the (b) LPGA and (c) control devices under varying bias voltages.

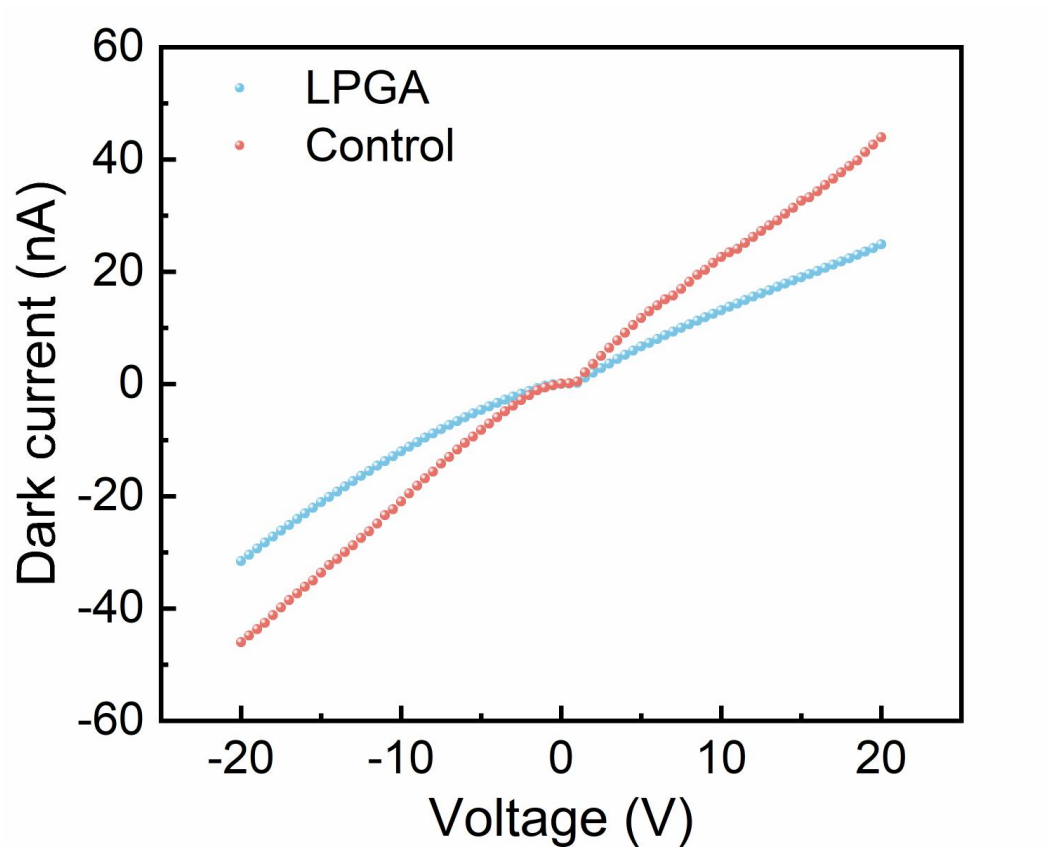

Figure S15. Dark current-voltage characteristics of the control and LPGA thick-film devices measured in the range of −20 to 20 V.

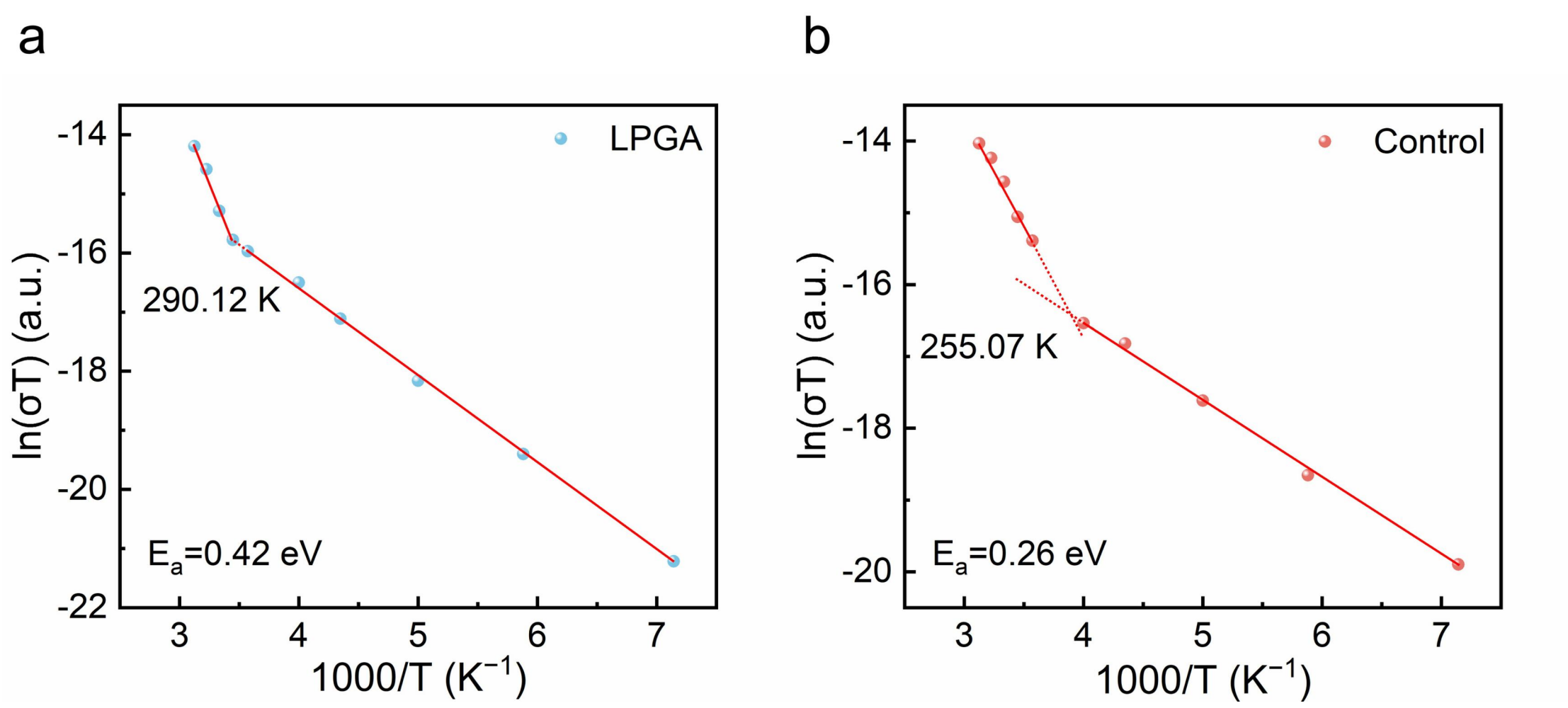

Figure S16. Temperature-dependent conductivity and Arrhenius plots of the (a) LPGA and (b) control devices.

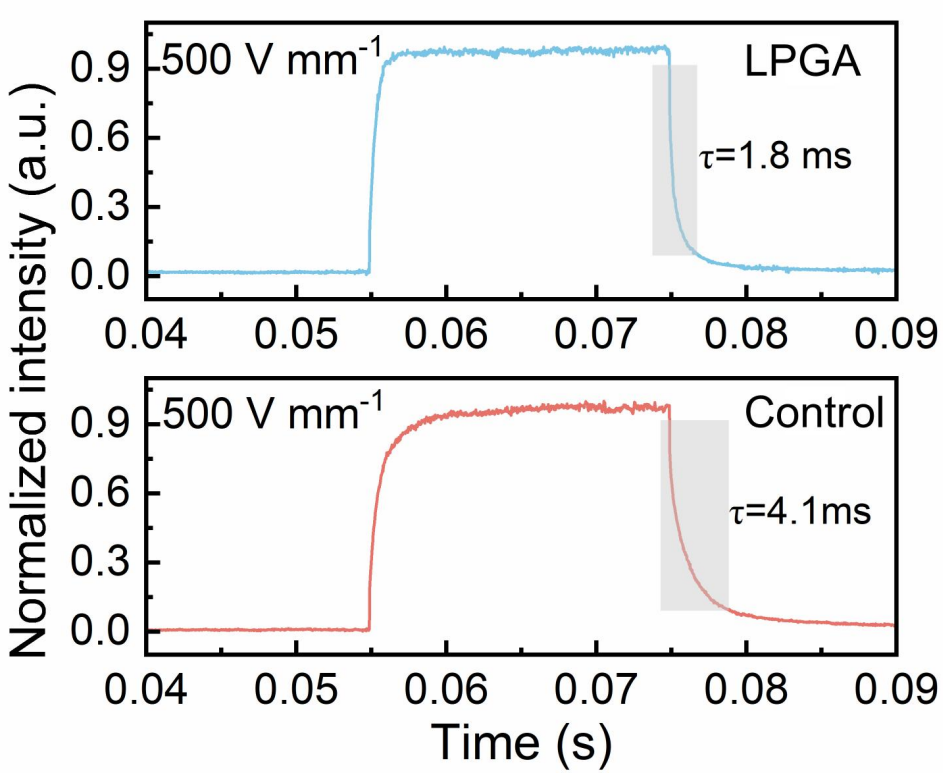

Figure S17. X-ray temporal response of the control and LPGA devices.

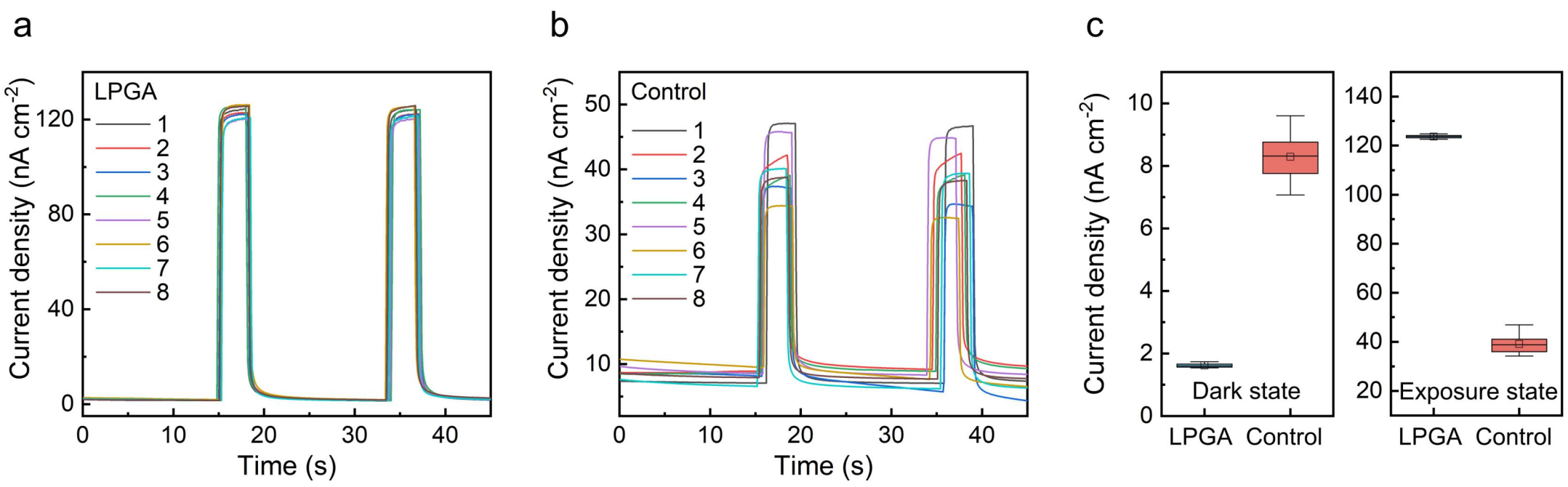

Figure S18. The spatial response across eight device regions. (a) LPGA device. (b) control device. (c) Box plots of dark current density and photocurrent density.

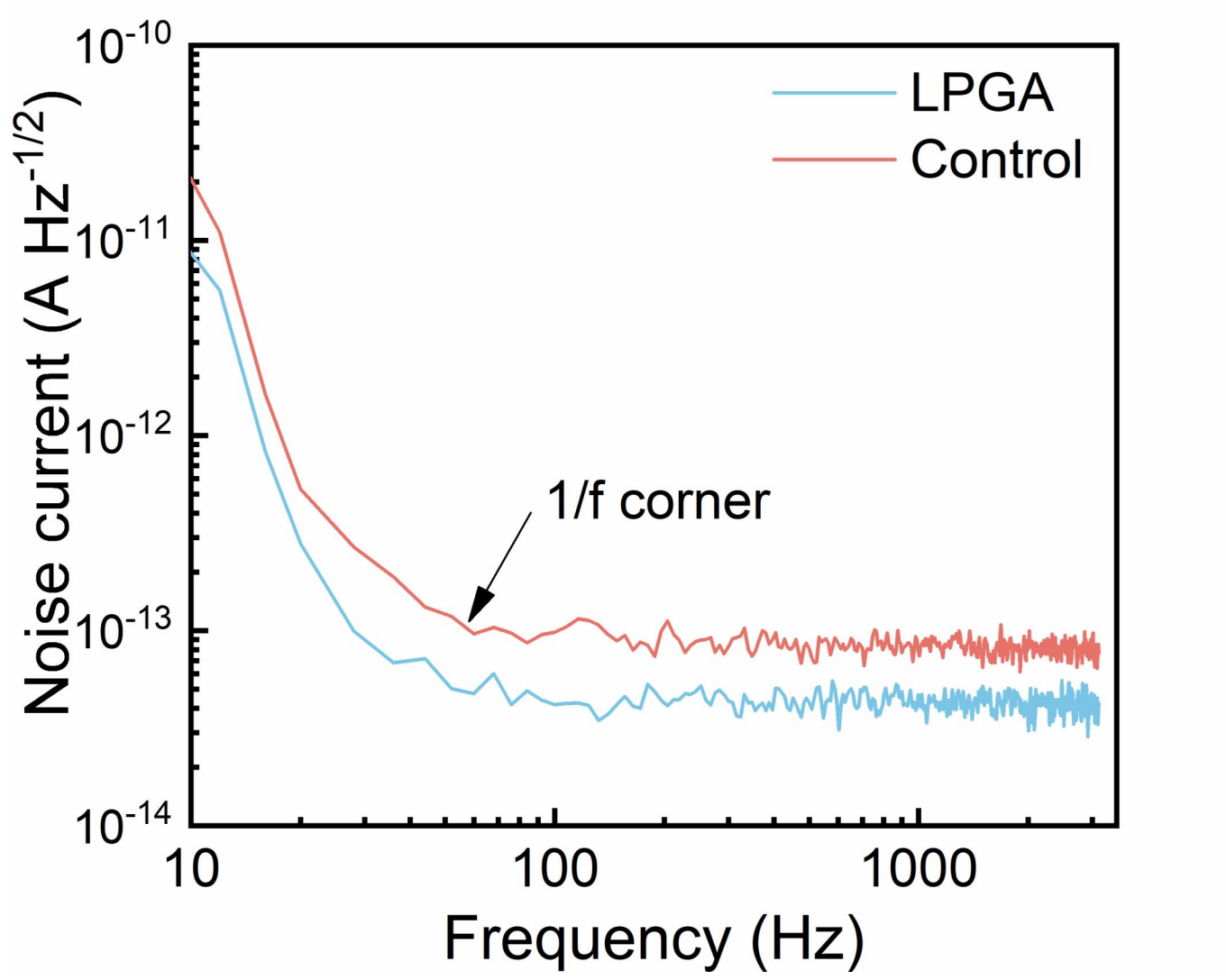

Figure S19. Noise current spectra of LPGA and control devices under zero dose rate.

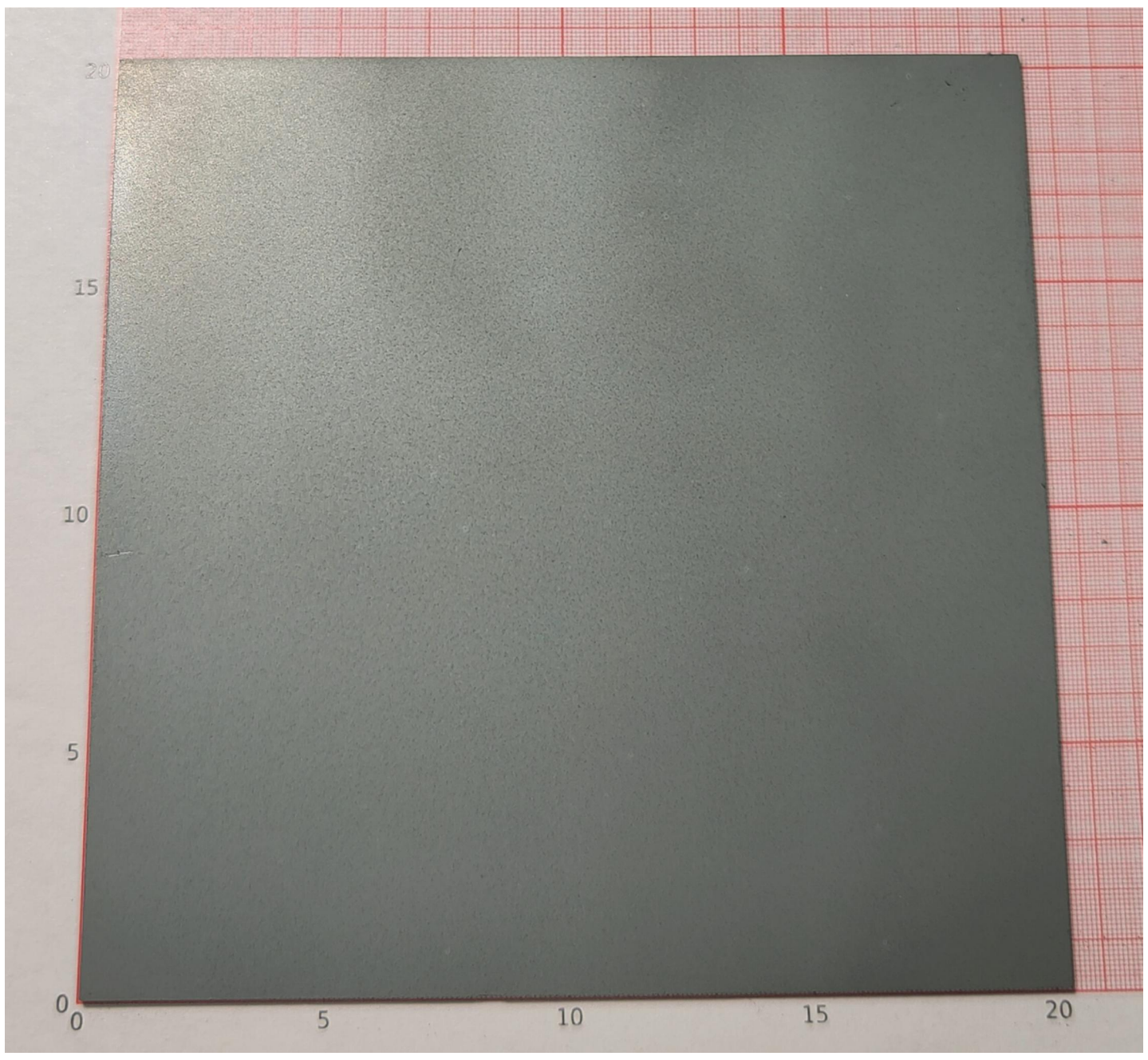

Figure S20. Optical photograph of large-area perovskite thick films prepared by liquid phase growth and annealing.

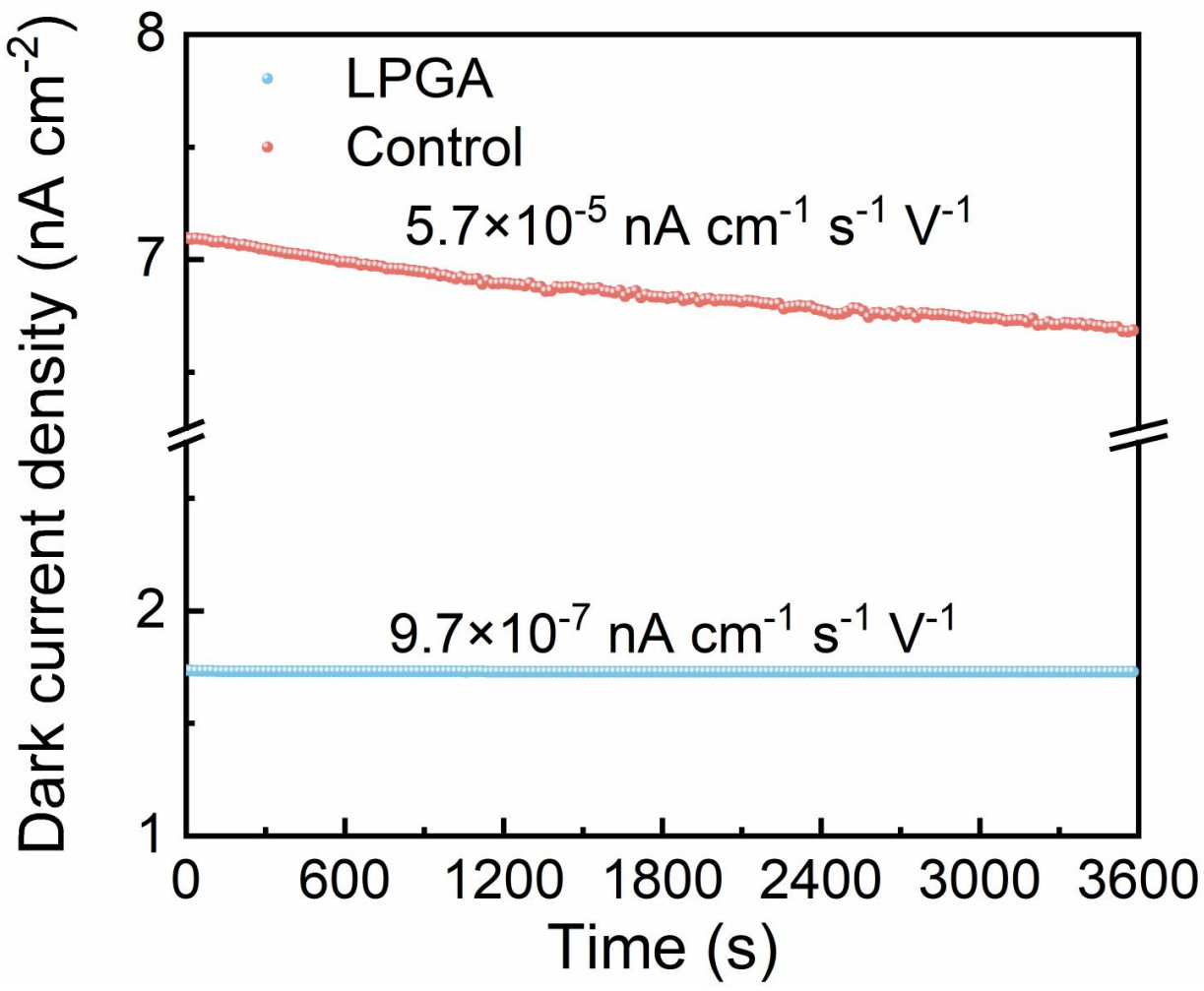

Figure S21. Dark current density variation of the control and LPGA devices over 3600 s.

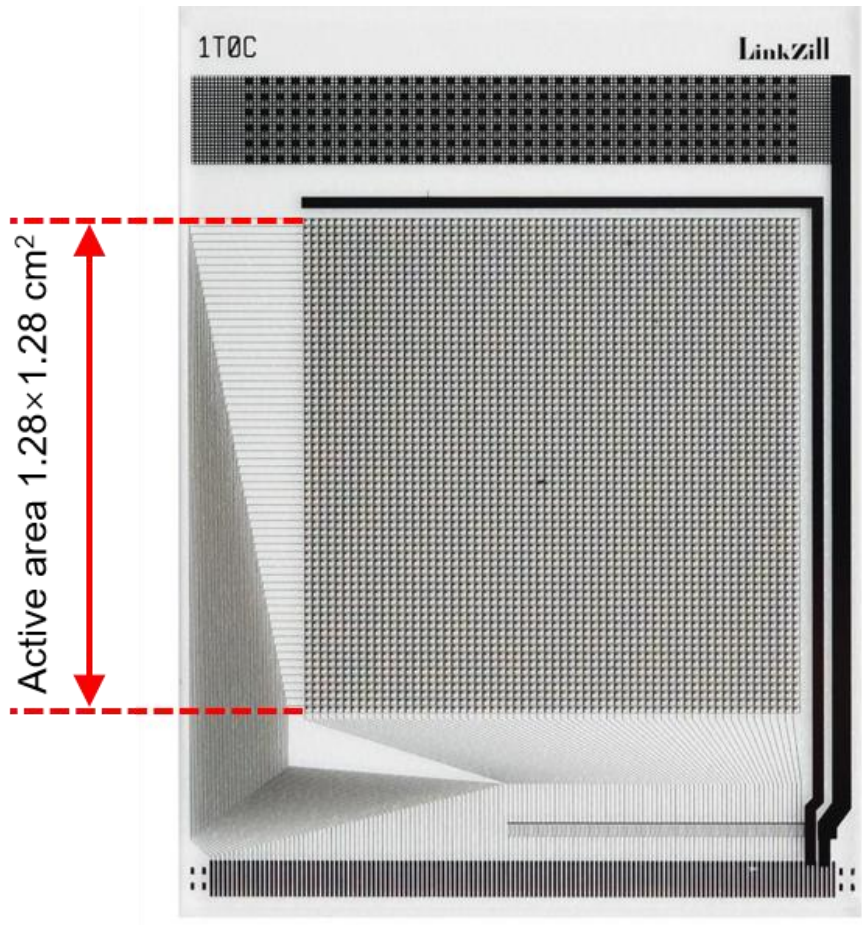

Figure S22. Optical image of the TFT chip.

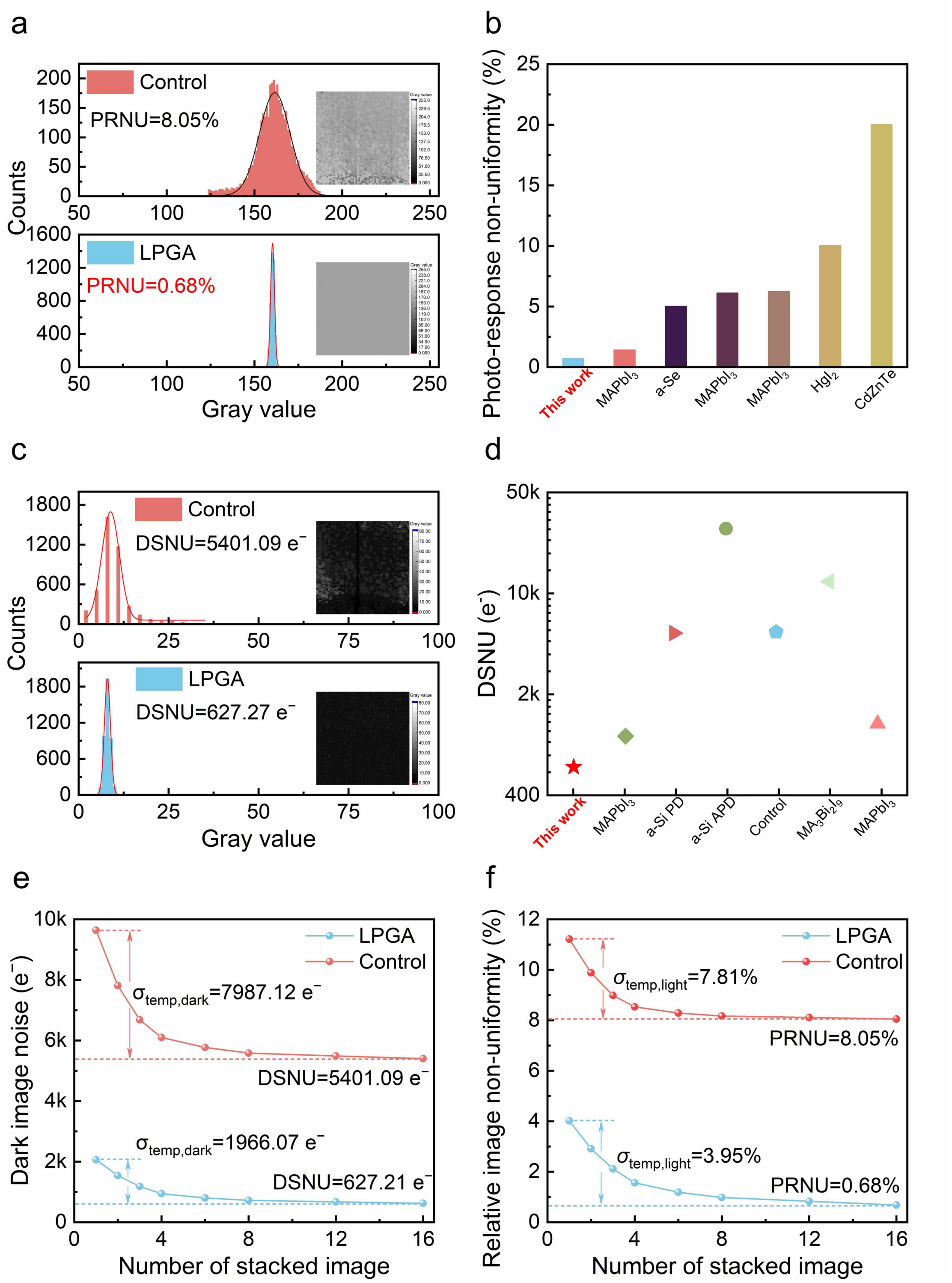

Figure S23. PRNU, DSNU, and stacked-frame decomposition of temporal and spatial noise. (a) Distribution of pixel grayscale values for all pixels of FPDs under X-ray exposure, with an inset showing the image at that grayscale value. (b) Comparison of the PRNU of representative X-ray FPDs. (c) Distribution of pixel grayscale values for all pixels of FPDs in the dark state, with an inset showing the image at that grayscale

value. (d) Comparison of the DSNU among representative image sensors. (e) Stacked-frame analysis of dark-state noise. (f) Stacked-frame analysis of illuminated-state non-uniformity.

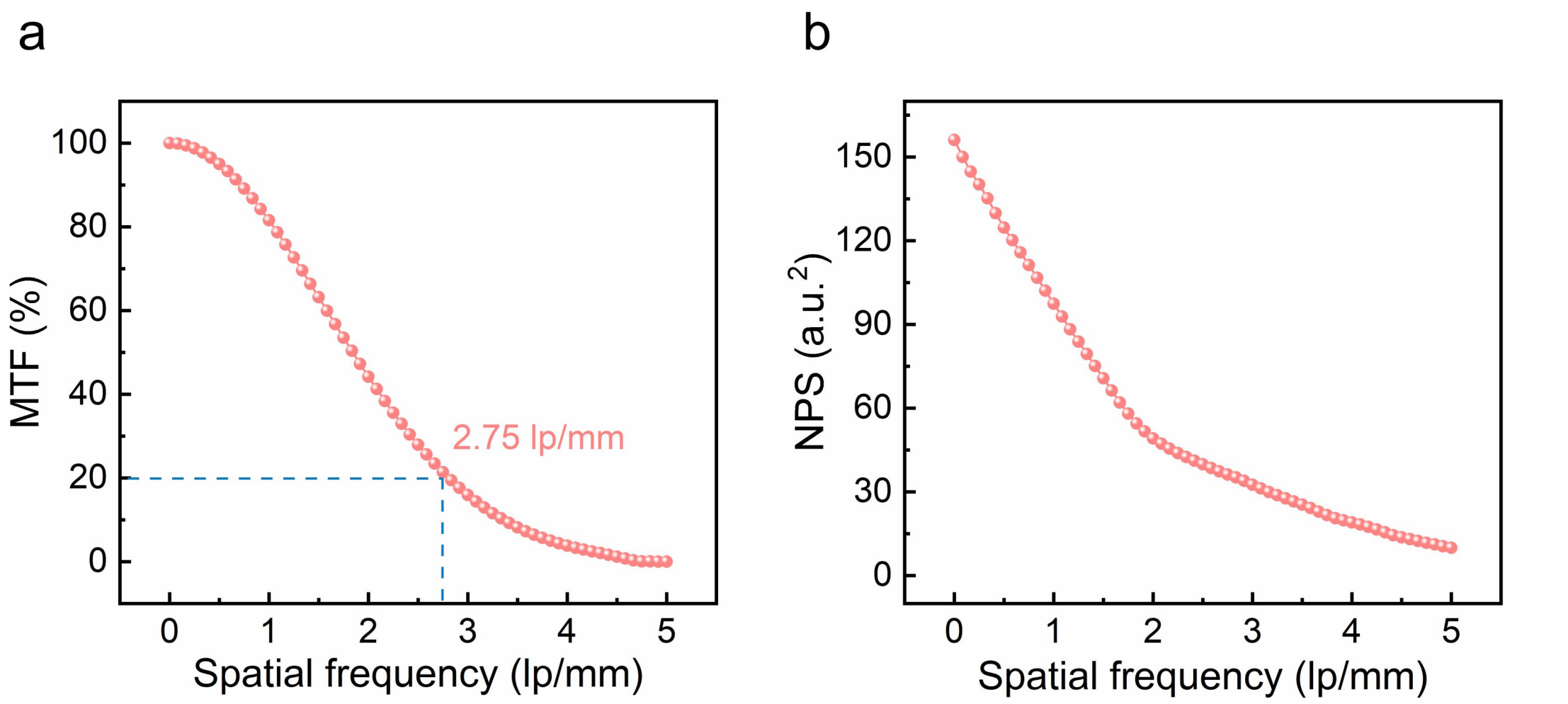

Figure S24. The derived MTF and NPS curves of the perovskite imager. (a) MTF. (b) NPS.

Table S1. The radiation quality of X-ray sources and corresponding parameters.

**Radiation Quality**	**Approximate X-ray tube voltage (kV)**	**Half-value layer (mm Al)**	**Additional filtration (mm Al)**	***SNR_{in}^2* (1/(mm²·µGy))**
RQA3	50	4.0	10.0	21759
RQA5	70	7.1	21.0	30174
RQA7	90	9.1	30.0	32362
RQA9	120	11.5	10.0	31077

Table S2. Material properties used for simulation.

	**Thermal conductivity ($W\ m^{-1}\ K^{-1}$)**	**Specific heat ($J\ kg^{-1}\ K^{-1}$)**	**Density ($g\ cm^{-3}$)**
Glass	1.4	750	2.5
Perovskite	0.3	306	4.2

Table S3. Performance comparison for benchmark X-ray flat panel detectors.

**Detector materials**	**Imaging dose (nGy_{air})**	**DQE@0 lp mm^{-1} (%)**	**Reference or models**

Semiconductors	$MAPbI_3$	25,000	N/A	*Nature* 2017, **550**, 87.
	$MAPbI_3$	4,170	N/A	*Nat. Electron.* 2021, **4**, 681.
	$MAPbI_3$	525,900	75.3	*Nat. Commun.* 2023, **14**, 6865.
	$CsPbBr_3$	260	N/A	*Nat. Commun.* 2024, **15**, 1588.
	$MAPbI_3$	5,800	N/A	*Device* 2025, **3**, 100617.
	CdTe	4,000	60	*Med. Phys.* 2022, **49**, 1481.
	CdTe	36,000	40	*IEEE T. Nucl. Sci.* 2002, **49**, 176
	a-Se	9,910	64	*Med. Phys.* 2017, **44**, 3965.
	a-Se	103,000	75	Fujifilm Amulet
	a-Se	15,130	38	Hologic DR-1000
	Si-strip	50,000	70	*Med. Phys.* 2007, **34**, 1918.
	GaAs	830	73	*Proc. SPIE* 10948.
	CdZnTe	36,000	65	*IEEE T. Nucl. Sci.* 2002, **49**, 176.
Scintillators	$CsPbBr_3$ QDs	15,000	N/A	*Nature* 2018, **561**, 88.
	Gd_2O_2S:Tb	148,347	36	*Med. Phys.* 2019, **46**, 4857.
	Gd_2O_2S:Tb	3,043	20	Varian PaxScan 4030
	CsI:Tl	54,695	69	*Med. Phys.* 2004, **31**, 2594.
	CsI:Tl	1,478	64	Varian PaxScan 2520
	CsI:Tl	2,347	64	GE Revolution XQ/i
	CsI:Tl	4,100	74	Dexela 2923
	BGO	220,000	19	*Med. Phys.* 2009, **36**, 5707.
	$BaFBr:Eu^{2+}$	2,608	29.5	Kodak CR-400/GP-25
**$FA_{0.5}MA_{0.5}PbI_3$**		**40.6**	**71.6**	**This work**

Table S4. Standard tissue weighting factors from International Commission on Radiological Protection.

**Tissue or organ**	**Tissue weighting factor (ω_T)**
Bone-marrow (red)	0.12
Colon	0.12
Lung	0.12
Stomach	0.12
Breast	0.12
Gonads	0.08

Bladder	0.04
Esophagus	0.04
Liver	0.04
Thyroid	0.04
Bone surface	0.01
Brain	0.01
Salivary glands	0.01
Remainder tissues* (13 total)	0.12 (collectively, each 0.01 individually)
